# A Single‐Cell Metabolic Profiling Characterizes Human Aging via SlipChip‐SERS

**DOI:** 10.1002/advs.202406668

**Published:** 2024-09-04

**Authors:** Fugang Liu, Jiaqing Liu, Yang Luo, Siyi Wu, Xu Liu, Haoran Chen, Zhewen Luo, Haitao Yuan, Feng Shen, Fangfang Zhu, Jian Ye

**Affiliations:** ^1^ School of Biomedical Engineering Shanghai Jiao Tong University Shanghai 200030 China; ^2^ State Key Laboratory of Systems Medicine for Cancer Shanghai Cancer Institute Ren Ji Hospital School of Medicine Shanghai Jiao Tong University Shanghai 200032 China; ^3^ Institute of Medical Robotics Shanghai Jiao Tong University Shanghai 200240 China; ^4^ Shanghai Key Laboratory of Gynecologic Oncology Ren Ji Hospital School of Medicine Shanghai Jiao Tong University Shanghai 200127 China

**Keywords:** aging, metabolic profiling, single‐cell, SlipChip, surface‐enhanced Raman spectroscopy

## Abstract

Metabolic dysregulation is a key driver of cellular senescence, contributing to the progression of systemic aging. The heterogeneity of senescent cells and their metabolic shifts are complex and unexplored. A microfluidic SlipChip integrated with surface‐enhanced Raman spectroscopy (SERS), termed SlipChip‐SERS, is developed for single‐cell metabolism analysis. This SlipChip‐SERS enables compartmentalization of single cells, parallel delivery of saponin and nanoparticles to release intracellular metabolites and to realize SERS detection with simple slipping operations. Analysis of different cancer cell lines using SlipChip‐SERS demonstrated its capability for sensitive and multiplexed metabolic profiling of individual cells. When applied to human primary fibroblasts of different ages, it identified 12 differential metabolites, with spermine validated as a potent inducer of cellular senescence. Prolonged exposure to spermine can induce a classic senescence phenotype, such as increased senescence‐associated β‐glactosidase activity, elevated expression of senescence‐related genes and reduced LMNB1 levels. Additionally, the senescence‐inducing capacity of spermine in HUVECs and WRL‐68 cells is confirmed, and exogenous spermine treatment increased the accumulation and release of H_2_O_2_. Overall, a novel SlipChip‐SERS system is developed for single‐cell metabolic analysis, revealing spermine as a potential inducer of senescence across multiple cell types, which may offer new strategies for addressing ageing and ageing‐related diseases.

## Graphical abstract

The SlipChip‐SERS system enables label‐free and sensitive profiling of single‐cell metabolism, offering a powerful tool to investigate cellular aging‐related metabolic alterations. Spermine is identified as a potential inducer of cellular senescence through single‐cell analysis, which illuminates metabolic changes in aging and advances the comprehension of the process of senescence mechanisms and therapeutic targets.

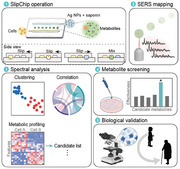

## 1 Introduction

Systemic aging is a complex, progressive phenomenon marked by a cascade of continuous multi‐level deteriorations within the organism.^[^ ^1^ ^]^ During aging, cells may undergo irreversible cell cycle arrest known as cellular senescence when exposed to specific acute stressors, such as metabolism dysregulation, direct DNA damage, oxidative stress, and telomere dysfunction.^[^ ^2^ ^]^ Senescent cells accumulate in various tissues during aging and contribute to the systemic aging process through the secretion of pro‐inflammatory and pro‐fibrotic factors, also known as senescence‐associated secretory phenotype (SASP).^[^ ^3^, ^4^ ^]^ Senescence is intricately linked to both intra‐ and extracellular metabolism, and specific metabolic alterations can effectively trigger this process. For instance, a diminished NAD^+^/NADH ratio (indicative of mitochondrial dysfunction), deviations from physiological oxygen levels, and hyperglycemia all emerge as pivotal inducers of cellular senescence.^[^ ^5^ ^]^ Moreover, senescent cells manifest distinctive metabolic shifts, characterized by the accumulation of free polyunsaturated fatty acids and transition metals, heightened activity in the sphingomyelin‐ceramide pathway, and depletion of dNTPs.^[^ ^5^, ^6^ ^]^ While metabolic reprogramming plays an essential role in sustaining the enduring cell cycle arrest of senescent cells, the intricate metabolic response to senescence and systemic metabolome alteration still needs further exploration.

Across diverse tissues, cells (even those within the same microenvironment) exhibit high heterogeneity in senescence level.^[^ ^7^ ^]^ Recent studies of systemic aging gradually focus on single‐cell levels to investigate the underlying cellular mechanisms during the aging process. The development of single‐cell transcriptomic atlases encompassing various tissues and age‐related diseases has greatly contributed to a comprehensive understanding of the cell‐specific mechanisms driving the aging process, revealing new senescence markers and potential intervention targets at single‐cell resolution.^[^ ^8^ ^]^ In comparison to single‐cell transcriptome analysis, single‐cell metabolomic analysis presents an opportunity to directly monitor real‐time metabolic status and provide a more comprehensive, stable, and intuitive understanding of physiological changes during cellular senescence. A growing body of literature is dedicated to exploring metabolic alterations during cellular senescence, with a recent study elucidating comprehensive lysosomal‐subpopulation‐specific metabolomic changes, thereby shedding light on the localized metabolomic responses contributing to senescence progression.^[^ ^9^ ^]^ Nevertheless, it's important to note that while the lysosomal metabolome provides valuable insights, it represents only a portion of the cellular metabolome. Alterations in metabolic patterns within mitochondria, the endoplasmic reticulum, and other cellular compartments involved in metabolic processes also play significant roles in driving cellular senescence. Therefore, interpreting the comprehensive cellular metabolomes during senescence is crucial for further elucidating the underlying mechanisms.

Emerging research has highlighted the significance of metabolic regulation in systemic aging, with mitochondrial dysfunction and dysregulation of nutrient‐sensing pathways identified as primary drivers of aging progression.^[^ ^4^ ^]^ Direct supplementation of specific metabolites can modulate cellular senescence. For example, N‐acetylcysteine has been shown to ameliorate cisplatin‐induced renal senescence by reducing the number of senescence‐associated β‐galactosidase (SA‐β‐gal) positive cells and decreasing the levels of p53 and p21.^[^ ^10^ ^]^ Additionally, various metabolites are implicated in the regulation of epigenetic modification enzymes, which control the expression of genes associated with cellular senescence.^[^ ^11^ ^]^ Polyamines, including putrescine, spermidine, and spermine, are natural metabolites involved in DNA replication, RNA transcription, protein synthesis, and post‐translational modification in all eukaryotic cells.^[^ ^12^ ^]^ Notably, the spermine level in whole blood altered during aging and the proportion of spermine in polyamines increased significantly with age, indicating a relevance between spermine metabolism and systemic aging.^[^ ^13^ ^]^ Additionally, the high level of putrescine, the precursor for spermine synthesis, has been observed in several age‐related diseases, such as cancer, Parkinson and ischemia disease.^[^ ^14^ ^]^ Previous studies also revealed higher level of N1‐acetylspermine, an intermediate in polyamine metabolism, was related to increased risk of all‐cause mortality and reduced odds of longevity.^[^ ^15^ ^]^ However, the underlying mechanisms of these alterations and whether these alterations contribute to the aging process remain unclear. Therefore, understanding cellular metabolism, such as polyamine metabolism, at the heterogeneous single‐cell level is important for unraveling the mechanisms of aging.

Due to the extremely low metabolite content at the single‐cell level, highly sensitive detection methods are required. Mass spectrometry (MS) is a prevalent technique for characterizing cellular metabolic profiles due to its multiple molecular profiling capabilities.^[^ ^16^ ^]^ However, MS‐based single‐cell analysis technique still face challenges including sensitivity to metabolites with small molecular weight, instrument cost, portability, and ease of use.^[^ ^17^ ^]^ In contrast, surface‐enhanced Raman spectroscopy (SERS), serving as a molecular fingerprint vibrational spectrum reflecting chemical bond information,^[^ ^18^ ^]^ offers non‐destructive, low cost, portable, and label‐free molecular detection capabilities even at the single‐molecule level via the enhancement of metallic colloids.^[^ ^19^ ^]^ SERS has made preliminary progress in the single‐cell detection of cellular membrane molecules,^[^ ^20^ ^]^ specific metabolites,^[^ ^21^ ^]^ disease‐related proteins,^[^ ^22^ ^]^ stress responses,^[^ ^23^ ^]^ and molecules within fixed cells,^[^ ^24^ ^]^ with less emphasis on label‐free, real‐time detection of intracellular metabolites. Furthermore, SERS application in single‐cell metabolomics from a global perspective remains limited, primarily due to the diverse diffusion and fluctuations of small metabolites in liquid systems, which makes it challenging to accurately and reproducibly capture the global metabolic profile of the sample solely through the traditional collection and analysis of single or averaged SERS spectra. Unlike these studies, we have recently concentrated on exploring intracellular multiple metabolites.^[^ ^25^ ^]^ By utilizing cell membrane perforation to release metabolites into the buffer solution, the point of analysis shifts from the cell itself to the metabolites secreted in solution. This approach not only circumvents interference from the cell membrane but also achieves uniformity in the liquid testing system and ensures test stability through the use of spectral set (i.e., SERSome) collections.^[^ ^26^ ^]^ The utilization of single‐cell SERS metabolomics analysis provides an opportunity to delve into the distinct SERS traits within heterogeneous aging cells. By conducting a comprehensive examination of characteristic peaks and validating the potential role of certain metabolites in cellular senescence through in vitro analysis, this approach contributes to uncovering and exploring senescence‐related metabolic pathways that serve as potential targets to ameliorate senescence.

In this work, we developed a SlipChip microfluidic system combined with the SERS technique, termed SlipChip‐SERS, for label‐free, high‐throughput, ultra‐sensitive detection and analysis of single‐cell metabolic profiles. In order to perform intracellular metabolic profiling at the single cell level, it generally requires compartmentalization of single cell first, followed by the release of intracellular metabolites and SERS analysis. This will require parallel manipulation of nanoliter droplets, which can be challenging for traditional droplet‐based microfluidic methods. The SlipChip microfluidic device can generate and manipulate liquid partitions in different volumes, typically from nanoliter to femtoliter, with simple slipping operations.^[^ ^27^ ^]^ We designed the SlipChip‐SERS device to enable multiple personalized operations on parallel droplets, including dispersion of single cells and metallic colloids, breaking the cell membrane and releasing intracellular metabolites, followed by subsequent Raman spectroscopy analysis. This system efficiently dispersed cells into enclosed nanoscale wells to form single‐cell droplets, and mixed the lysate to release intracellular metabolites. By collecting the SERSomes of single‐cell metabolites, we were able to sensitively, reliably, and conveniently resolve single‐cell metabolic fingerprints, achieving cell clustering. Initially, we applied the system to isolate individual cancer cells, and the corresponding metabolic profiling analysis verified the robustness of the technique. Subsequently, we examined the single‐cell metabolic profiles of young and aged human fibroblast cells with this system to identify potential senescence markers. Further, we used a two‐step metabolites screening to narrow the aging‐promote metabolite candidates and identified the most potential metabolite, spermine, on the cellular level. We concluded that spermine could induce cellular senescence in various cell types in vitro, providing an opportunity to target the spermine‐related pathway to intervene in cellular senescence. Therefore, our SlipChip‐SERS system provides a powerful tool for the in‐depth study of single‐cell metabolism, which is expected to play an important role in biomedicine and drug discovery.

## 2 Results and Discussion

### 2.1 Development of the SlipChip‐SERS System

**Figure 1:**
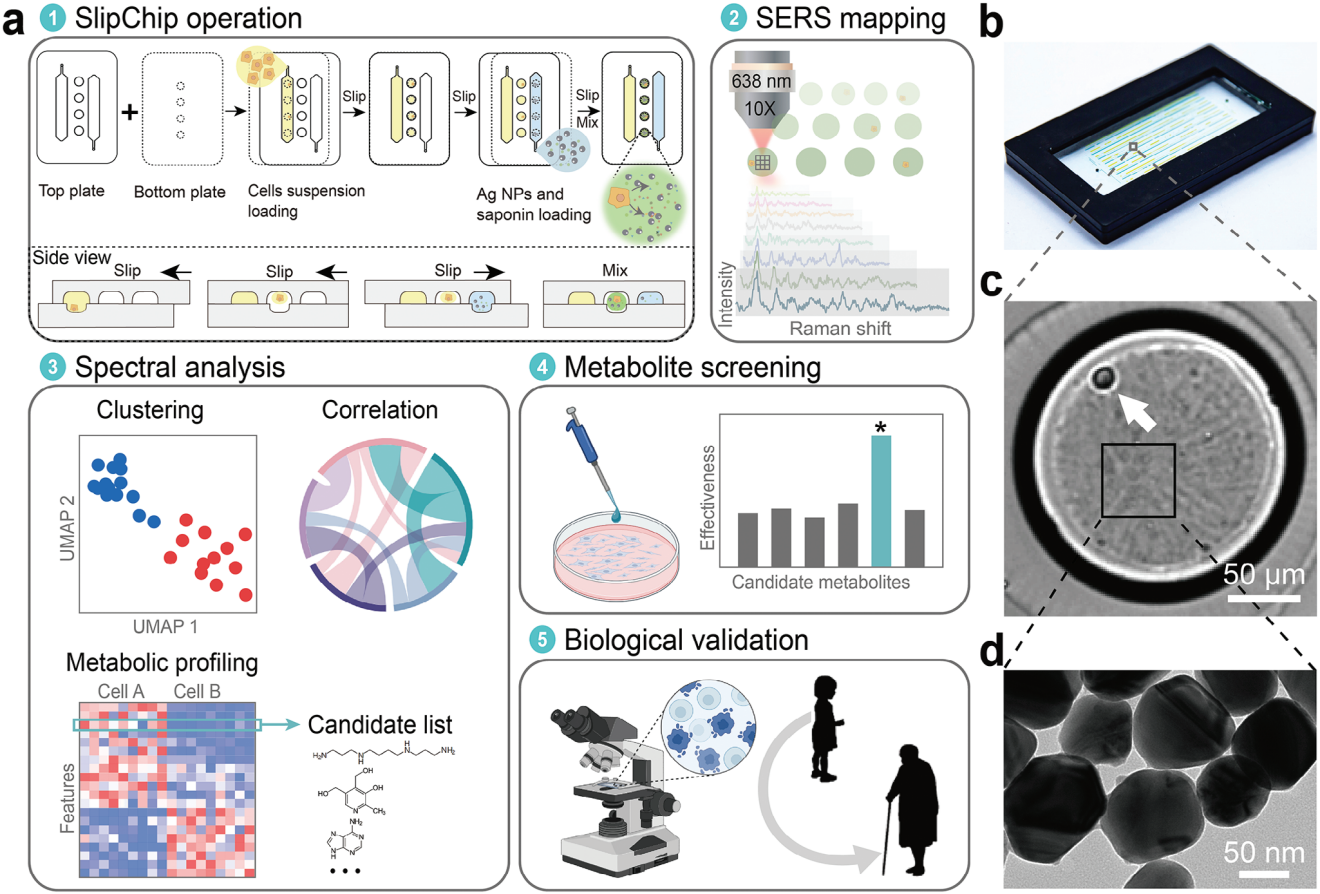
The detection of metabolic profiling at single‐cell level via SlipChip‐SERS microfluidic platform. a) The experimental procedure includes five steps. 1) The SlipChip was operated to inject and mix the cellular suspension and silver (Ag) nanoparticles (NPs) with saponin, which broke the cell membrane to release intracellular metabolites. 2) The microwells containing a single cell were objected under the microscope and a SERS spectral set was collected. 3) Analyze the single‐cell population spectral set acquired, gaining the candidate metabolites. 4) Candidate metabolites were under screening to see their biological effectiveness. 5) Further biological functional validation was performed. b) A photograph of a SlipChip device. c) The bright‐field image of a microwell with a single MCF‐7 cell indicated by a white arrow. d) A TEM image of Ag NPs dispersed in the microwell.

We developed the SlipChip‐SERS system for single‐cell metabolic profiling and analysis primarily because of its advantages including a convenient step‐by‐step approach for single cell partitioning, cell membrane disruption, and metallic colloid loading (**Figure** **1**a). The SlipChip device consists of two plates, top and bottom, fabricated from quartz to minimize background signals in the SERS measurements. The top plate contains the fluidic loading channels and an array of large receiving microwells; the bottom one contains an array of transporting microwells. The surfaces of both plates were silanized to be hydrophobic and a lubricating oil consisting of tetradecane and silicone oil was placed between the two plates during device assembly. The typical workflow for the single‐cell metabolism study using SlipChip‐SERS consists of the following steps. **(1) Single‐cell isolation in the SlipChip**: first, the cell‐loading fluidic channel on the top plate is aligned with the transporting microwell on the bottom plate. The cell suspension with an appropriate concentration is then introduced into the device via pipetting, ensuring that a certain amount of single‐cell microwells are formed according to Poisson statistics on the bottom plate. Next, the top plate is slipped against the bottom plate to allow the large receiving microwells on the top plate in alignment with the transporting microwells on the bottom plate. Due to the imbalance of capillary pressures created by non‐equal feature dimensions in the bottom and top of the microwell, the aqueous droplets in the transporting microwells are driven into the receiving microwells.^[^ ^28^ ^]^ Mixing of single cells and nanoparticles (NPs) in the SlipChip: the top plate is then slipped against the bottom plate to align the second loading channel on the top plate with the transporting microwells on the bottom plate, and the aqueous reagent containing metallic colloids and a cell membrane‐breaking agent is introduced into the device. Finally, the top plate is slipped back to align the receiving microwells on the top plate with the transporting microwells on the bottom plate, and the two kinds of droplets are merged rapidly so that the droplets encapsulating the cell lysate are formed. **(2) SERS testing**: after generating single‐cell droplets and fulfilling cell lysis in the SlipChip platform, we acquire SERS spectral sets from droplets containing single cells identified under microscopic examination. During this phase, metabolites released from the perforated cells are captured within the hotspots of the metallic colloids. **(3) Data analyses**: using the SERS spectral sets of single‐cell populations, we perform a series of data analyses, including cluster analysis, correlation analysis, metabolic profiling, and the acquisition of candidate metabolites with potentially important roles of interest. Candidate metabolites are screened by comparing the cell spectral sets with the spectra of common pure metabolites. **(4) Screening of metabolic biomarkers**: in vitro experiments are conducted at the cellular level to screen candidate metabolites. Candidate metabolites are separately introduced into cell cultures to characterize changes in cell growth, and specific metabolites that significantly impact cellular physiology will be identified. **(5) Further validation of target metabolites**: the hit metabolites undergo further validation. More comprehensive assays, such as post‐cell culture staining with classical markers to characterize cellular states, are conducted to verify the effects of target metabolites on cells and elucidate their significant biological implications.

A photograph of a typical SlipChip device with 768 microwells filled with food dyes is shown in Figure 1b. The workflow and the arrays of droplets generated in different steps were characterized and visualized with yellow and blue dyes (Figure S1a,b, Supporting Information). The bright‐field image was captured by the microscope and the statistical distribution of droplet size proves that the droplet formation is stable and uniform (Figure S1c, Supporting Information). The microwell with a typical diameter of 260 µm contains a 2.8 nL aqueous droplet (Figure 1c), where the white arrow indicates a single human breast cancer (MCF‐7) cell as an example and the black square shows the silver (Ag) colloids of approximately 80 nm in size uniformly dispersed in the droplet (Figure 1d). The SlipChip‐SERS microfluidic platform shows great potential as a tool for both basic and clinical research, due to several feature that come with this system. First, we have integrated single‐cell separation, lysis, and mixing on the SlipChip microfluidic device. This eliminates the need for complex, large instruments and avoids the typical low‐throughput operation of the 96‐well plate. The two‐step sample loading process reduces the risk of premature cell damage, thereby preserving the original metabolic state to a greater extent. Additionally, the confinement of cells within nanoliter‐scale droplets enhance the concentration of metabolites at the single‐cell level. The minimal volume requirements for the microfluidic device are particularly advantageous for the handling of precious samples. Since the cell membrane has been disrupted and intracellular metabolites are released, the droplet contains metallic colloids and abundant metabolites. Here, the metallic colloids with varying materials, sizes, and surface charges can be chosen to fit different purposes. The reproducibility of liquid‐phase detection could be overcome through statistical methods, reducing the fluctuations in individual spectra arising from the random Brownian motion of NPs and metabolites within the droplet and the different amounts of plasmonic hotspots across various test spots.^[^ ^26^, ^29^ ^]^ Hence, we acquired a set of fluctuating SERS spectra, i.e., SERSome,^[^ ^26^ ^]^ in the single droplet to depict the metabolic profile of a single cell for subsequent metabolic analysis. Subsequently, we employ a non‐destructive SERS assay, enabling in situ and sensitive analysis of single‐cell metabolites. While this platform currently generates only 768 droplets at a time, limiting single‐cell analysis capacity, as previous studies have demonstrated that the SlipChip can simultaneously produce tens of thousands of droplets for gene^[^ ^30^ ^]^ and protein^[^ ^31^ ^]^ analyses.

### 2.2 Single‐Cell Metabolic Analysis of Cancer Cell Lines

To demonstrate the feasibility of single‐cell metabolic profiling using the SlipChip‐SERS system, we selected the MCF‐7 cells line as a representative model due to its widespread use as a common tumor cell line in single‐cell analysis. Based on the Poisson distribution rule for cell encapsulation within droplets,^[^ ^32^ ^]^ we determined an optimal cell loading concentration of 1–2 × 10^6^ mL^−1^, which facilitates the reliable single‐cell encapsulation events (Figure S2, Supporting Information). For the SERS substrate, we utilized citrate‐reduced Ag NPs at 0.2 nM (as synthesized), with negatively charged surfaces, due to their excellent stability and exceptional Raman enhancement performance, enabling detection down to the single‐molecule level.^[^ ^33^ ^]^ These Ag NPs exhibited an extinction peak at 419 nm (Figure S3c, Supporting Information), a hydrodynamic diameter of 78.1 ± 0.7 nm with a polydispersity index (PDI) of 0.248, and a zeta potential of −38.7 mV (Figure S3d, Supporting Information). The citrate‐coated Ag NPs were selected as surface‐accessible plasmonic‐enhancing substrates,^[^ ^34^ ^]^ to facilitate the adsorption and detection of a wide range of metabolites via the electrostatic interaction, covalent bonding and so on.^[^ ^35^, ^36^ ^]^ For cell membrane disruption, we employed saponin. Compared to other common membrane permeabilizers like sodium dodecyl sulfate (SDS) and TritonX‐100, which form larger pores by dissolving lipids or proteins in the cell membrane, saponin is milder in its damage. It effectively creates nanoscale pores with a diameter of approximately 10 nm by solubilizing cholesterol present in the cell membrane.^[^ ^37^ ^]^ Saponin also exhibits minimal SERS background interference (Figure S3a, Supporting Information) and yields superior metabolite signals (Figure S3b, Supporting Information) compared to conventional cell lysis reagents. Importantly, saponin does not compromise the stability of the Ag NPs, as confirmed in Figure S3c,d (Supporting Information). The SERS signal, following the adsorption of metabolites on Ag NPs, gradually increased within the first 60 min. Subsequently, during the test time window from 60 to 120 min, the SERS signals demonstrated relative stability (Figure S3e,f, Supporting Information). Therefore, the SlipChip device was allowed to equilibrate at room temperature for 60 min upon completion of sample encapsulation. Due to the stochastic nature of the Brownian motion of both metabolites and Ag NPs within the droplets, fluctuations in the collection of a single SERS spectrum are expected.^[^ ^25^ ^]^ To address this, a SERSome was acquired to capture the global metabolic profile of each single cell. We utilized the Pearson correlation coefficient (PCC) to evaluate the SERSome stability and determine the minimum number of spectra for a single SERSome. Our findings indicated that a 36‐spectrum test was sufficient, confirmed by a PCC value above 0.999 (Figure S4, Supporting Information). The designated region for SERSome acquisition for each single cell was a 50 µm × 50 µm extracellular area, with a minimum distance of 5 µm from the cells to avoid directly capturing signals from them (Figure S5, Supporting Information). The metabolites demonstrated a stable distribution within the droplet, as evidenced by the averaged SERS spectra and the mapping measurements across different locations in the droplet due to diffusion effects.^[^ ^38^ ^]^

**Figure 2:**
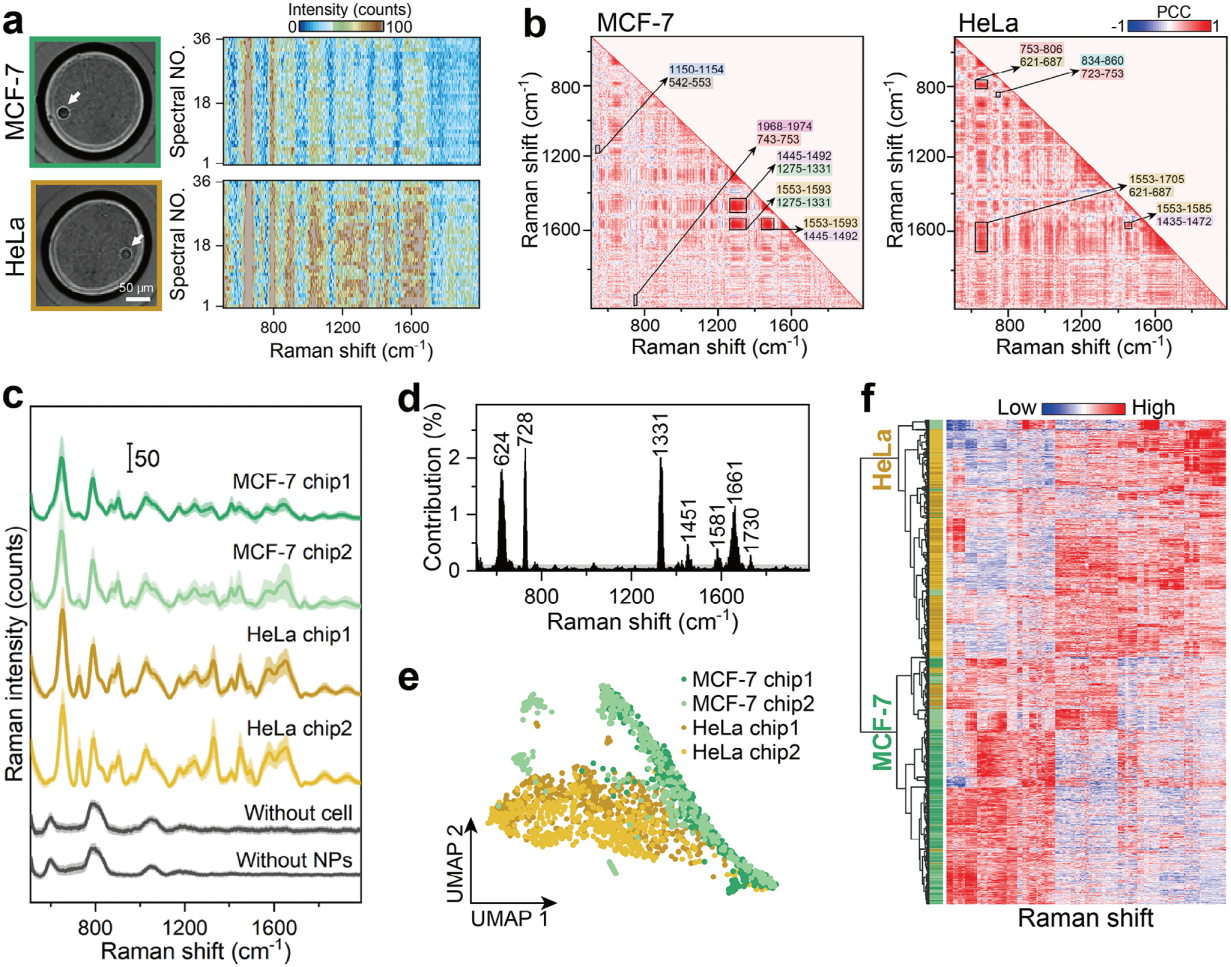
Discrimination of cancer single‐cell populations. a) Representative optical images and heat maps of SERS spectral sets of a single MCF‐7 cell and HeLa cell. White arrows point to the single cells. b) The global features’ correlation matrix of the SERS spectral set in (a), forms a metabolic signature and reveals the degree of dynamics for metabolite conversions. Highly positive/negative correlated SERS band pairs were marked with boxes and arrows pointing to the corresponding Raman shifts. c) Averaged spectra of MCF‐7 cells measured in chip 1 (n = 13) and chip 2 (n = 13), HeLa cells in chip 1 (n = 14) and chip 2 (n = 14), and background (microwells without either NPs or cells). The shaded areas indicate the standard deviation. d) The discriminating Raman shift for two cancer cell lines was screened out (contribution > 0.1%) to classification by random forest. e) UMAP plots of four kinds of samples using discriminating Raman shift in (d) of all spectral sets. The results of different batches of experiments were comparable. f) Hierarchical clustering heatmap of the discriminating Raman feature profiles. The color map shows the relative intensity with Raman shifts.

To demonstrate the metabolic analysis capability of this technique for different single‐cell populations, we selected two cancer cell lines: MCF‐7 cells and human cervical cancer (HeLa) cells as representatives in this study. Bright‐field images of typical microwells confirm the formation of a single cell suspension in the droplet, and their corresponding heatmaps of SERSomes responded to the metabolic state of each cell (**Figure** **2**a). For example, both cells have strong SERS peaks near 650 cm^−1^, attributed to C–S stretching present in numerous metabolites.^[^ ^39^ ^]^ However, SERS peaks associated with C–C stretching (1077 cm^−1^),^[^ ^40^ ^]^ ring‐breathing modes of adenine (Ade) and guanine (Gua) (1320 cm^−1^),^[^ ^41^ ^]^ and COO^−^ stretching (1560 cm^−1^),^[^ ^42^ ^]^ among others, are notably stronger in HeLa cells (see selective pure metabolite SERS spectra in Figure S6 (Supporting Information) and descriptions in Table S1, Supporting Information). We further analyzed the PCC matrices of the two single‐cell spectral sets, revealing the dynamic degree of peak transformations within the SERS spectra, with values ranging from −1 to 1^[^ ^43^ ^]^ (Figure 2b). Given that SERS spectra serve as metabolite fingerprint spectra, the peak correlations reflect chemical bond correlations of metabolites. Specifically, a PCC value close to 1 (red) indicates that the intensity changes of two peaks follow the same trend, potentially indicating related metabolites within the same metabolic network or different vibrational modes of the same metabolite. Conversely, a PCC value near −1 (blue) suggests that the intensity changes of two peaks exhibit opposite trends, potentially indicating associations between upstream and downstream metabolites. For example, a strong positive correlation is observed between 1450 and 1560 cm^−1^ SERS bands in both cells, attributed to the simultaneous occurrence of CH_2_ deformation and COO^−^ stretching vibrational modes in metabolites like organic acids and amino acids.^[^ ^39^, ^42^ ^]^ The appearance of the 1150 cm^−1^ block in MCF‐7 cells can be related to the up‐regulation of glutamine (Gln) metabolism in breast cancer cells.^[^ ^44^ ^]^ Its negative correlation with the 542 cm^−1^ (S–S stretching of cystine, Cys) band may be due to the involvement of cystine in promoting the catabolism of Gln.^[^ ^45^ ^]^ Noteworthy correlations are observed in HeLa cells at 650 and 753 cm^−1^ bands, indicative of multiple purine signals, possibly associated with the unusual purinergic signaling in cervical cancer cells.^[^ ^46^ ^]^

We further performed batch experiments on the two cell lines, wherein the average spectra of the same cells in the two chips were comparable, the spectra of different types of cells were distinguishable, and the cell spectra exhibited distinct metabolite SERS peaks compared to the background spectra (Figure 2c). To differentiate between the two types of cell lines, we employed random forest to assess the contribution of Raman shifts to the classification, thereby identifying important classification features such as 624 (CC twist), 728 (Ade), and 1331 cm^−1^ (CH deformation)^[^ ^39^, ^42^ ^]^ (Figure 2d). Notably, we used the sets of spectra of all cells for classification rather than the common averaging of spectra, as this approach allows for higher signal‐to‐noise ratios for differential features (Figure S7, Supporting Information). Leveraging these important SERS features, we performed data dimensionality reduction using uniform manifold approximation and projection (UMAP) to visualize the separation of the data, where spectra of different cells can be clearly separated and spectra of the same cells show repeatability (Figure 2e). Additionally, we clustered the features using hierarchical clustering, resulting in separate clusters for HeLa and MCF‐7 cells, with distinctly different Raman shifts reflecting the distinct metabolic profiles of the two cell types (Figure 2f). Therefore, the SERS‐SlipChip system was successfully designed to detect metabolite features of different cell types at the single cell level.

### 2.3 Application of Single‐Cell Metabolic Assay for Aging Analysis

**Figure 3:**
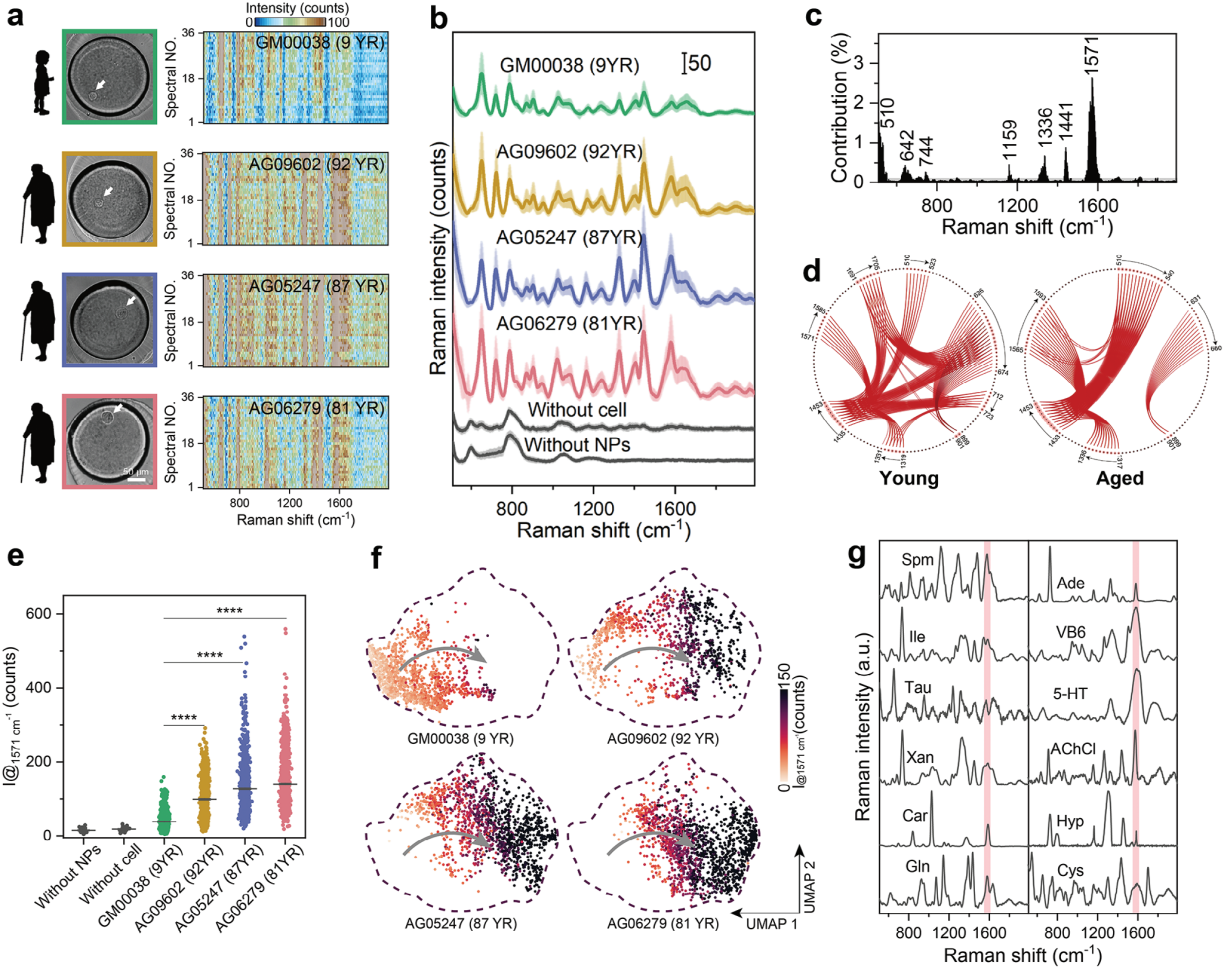
Application of discrimination between young and aged single‐cell populations. a) Optical images and heat maps of SERS spectral sets of representative single young and aged cells, white arrows point out the single cell. b) Averaged spectra of GM00038 single‐cells (n = 39), AG09602 single‐cells (n = 31), AG05247 single‐cells (n = 41), and AG06279 single‐cells (n = 46), and background (microwells without either NPs or cells). The shaded areas indicate the standard deviation. c) The discriminating Raman shift for young/aged cell lines was screened out (contribute > 0.1%) to classification by random forest. d) The discriminating Raman band correlation of young and aged single‐cells (ρ > 0.8, P < 0.05). e) The intensity distribution of spectra of background, young and aged single‐cells at 1571 cm^−1^ (potential aging indicator). Statistical significance was calculated using a two‐tailed *t*‐test (*p < 0.05; **p < 0.01; ***p < 0.001; ****p < 0.0001). f) All single cells are colored by the SERS intensity of 1571 cm^−1^. UMAP plots compare the young cells with the aged cells, the arrows show the migration trajectory from young to aged. g) SERS spectra for the candidate list of aging markers, the potential senescence indicator (1571 cm^−1^) was marked by yellow bands (see the abbreviations of metabolites in Table S1, Supporting Information).

Encouraged by the powerful resolving ability for different cell lines, we further applied this SlipChip‐SERS system to the single‐cell metabolic detection of fibroblasts with different senescence levels. We collected the SERSomes (**Figure** **3**a,b) of single cells from primary young (GM00038, 9 YR, 39 cells) and aged (AG09602, 92 YR, 31 cells; AG05247, 87 YR, 41 cells; AG06279, 81 YR, 46 cells) fibroblasts isolated from healthy individuals, which are widely used in aging‐related research^[^ ^47^ ^]^ (Figure S8, Supporting Information), and extracted the SERS differential features of young and aged cells. Cluster analysis divided the cells into two groups based on their senescence status (Figure S9, Supporting Information). The SERS differential features included peaks at 510 cm^−1^ (S–S stretching), 1441 cm^−1^ (CH_2_ deformation), 1571 cm^−1^ (C–N, C–C stretching), among others,^[^ ^45^, ^48^ ^]^ with the 1571 cm^−1^ peak being particularly notable for its role in separating the young and aged cells (Figure 3c). Additionally, PCC analysis (Figure 3d) revealed that young cells had many more correlated peak pairs compared to aged cells, possibly reflecting metabolic alterations during aging.^[^ ^4^ ^]^ In contrast, aged cells showed increased correlation only within specific peak pairs, notably the peak at 1571 cm^−1^, which contributed significantly to the classification. Thus, the 1571 cm^−1^ peak was speculated to indicate metabolites that significantly regulate aging pathway. Among the statistical results of the four cell lines, the intensity of the 1571 cm^−1^ peak was significantly higher in all three aged cell types than in young cells (Figure 3e). Dimensionality reduction of single‐cell spectra using UMAP also demonstrates the partitioning of cells and the distribution of 1571 cm^−1^ peak intensity as an aging feature (Figure S10, Supporting Information). The senescence feature intensity of young cell spectra was generally lower, while that of aged cells was predominantly higher, as shown in Figure 3f. Notably, there was heterogeneity in the spectra of the same type of cells, which can be seen in the migration trajectory from young to aged. For instance, in the UMAP of young single cells, although the intensity of the peak at 1571 cm^−1^ in most spectra is relatively low, a subset of spectra shows a tendency to migrate toward the region indicative of aging. This suggests that within the young cell population, some individual cells exhibit characteristics of aging, highlighting the heterogeneity of cellular aging at the single‐cell level. Based on these results, we searched pure metabolite spectra we discovered in our lab^[^ ^26^ ^]^ and identified 12 metabolites with 1571 cm^−1^ characteristic peak (Figure 3g), i.e., spermine (Spm), isoleucine (Ile), taurine (Tau), xanthine (Xan), L‐Carnosine (Car), L‐Glutamine (Gln), adenine (Ade), pyridoxine (VB6), serotonin (5‐HT), acetylcholine chloride (AChCl), hypoxanthine (Hyp), and cystine (Cys), and then performed further biological screening to verify which metabolite plays a key role in the regulation of aging.

### 2.4 Screening for Aging‐Promoting Metabolites at the Cellular Level

**Figure 4:**
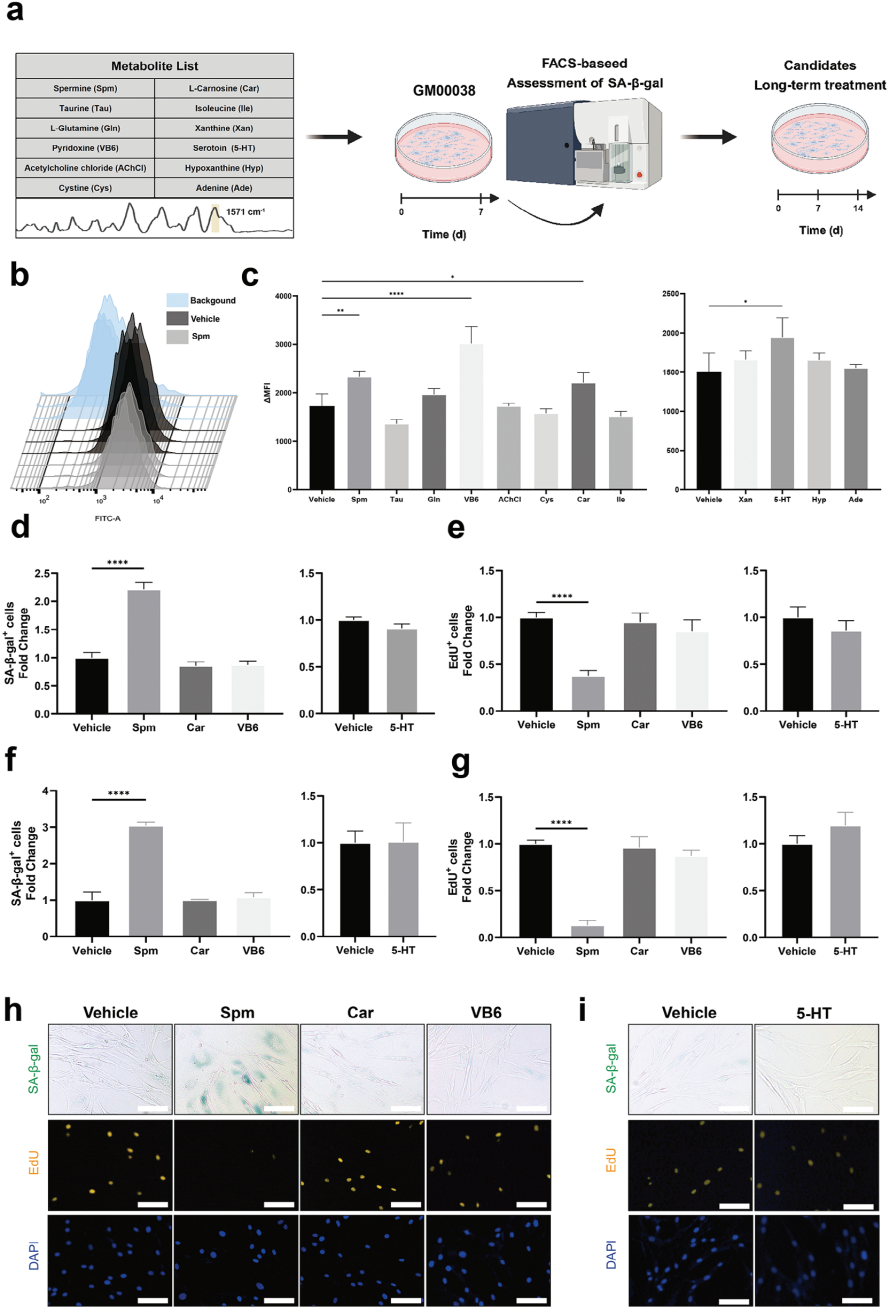
Screening for aging‐promoting metabolites based on SERS features. a) Schematic outlining of the design of aging‐promoting metabolites screening based on SERS features. GM00038 cells were treated with 12 metabolites separately for 7 days and then the SA‐β‐gal level was evaluated with FACS. Potential candidates were further assessed by SA‐β‐gal and Edu staining during long‐term treatment in vitro. b) Representative SA‐β‐gal signal quantified by FACS. ΔMFI = MFI of each sample—average MFI of background. c) Initial screening for aging‐promoting candidates among 12 metabolites. Water as solvent group was on the left and DMSO as solvent group was on the right. n = 3 biological replicates. d,e) Quantification of SA‐β‐gal^+^ cells (d) and EdU^+^ cells (e) after 7‐day treatment of potential candidates. n = 3 biological replicates. At least 300 cells per n were calculated. f,g) Quantification of SA‐β‐gal^+^ cells f) and EdU^+^ cells (g) after 14‐day treatment of potential candidates. n = 3 biological replicates. At least 300 cells per n were calculated. h,i) Representative images of SA‐β‐gal, EdU (yellow) and DAPI labeled nuclei (blue) of 14‐day candidates screening. Water solvent group (h) and DMSO solvent group (i). Scale bar, 100 µm. Data are means ± SD of biologically independent samples. Statistical significance was calculated using an ordinary one‐way ANOVA (*p < 0.05; **p < 0.01; ***p < 0.001; ****p < 0.0001).

To elucidate the potential aging‐related function of metabolites based on the 1571 cm^−1^ characteristic peak, we performed a two‐step metabolite screening in young human fibroblast (GM00038), a primary cell type with relatively low aging‐related background features (**Figure** **4**a; Figure S11, Supporting Information). The preliminary screening lasted for 7 days and the function of candidate metabolites was determined through the senescence‐associated β‐galactosidase (SA‐β‐gal) activity measured by flow cytometry (Figure 4b; Figure S16, Supporting Information). Considering the impact of metabolites treatment on cells was sustained but relatively mild, the secondary screening lasted for 14 days to verify the long‐term treatment effect, and traditional SA‐β‐gal and 5‐ethynyl‐2′‐deoxyuridine (EdU) staining used to validate the specific change on the cellular level, either on the 7th day or 14th day (Figure 4a).

After 7‐day metabolite treatment, cells incubated with 4 metabolites (Spm, VB6, Car, and 5‐HT) exhibited significant elevation of SA‐β‐gal activity and these candidates were used for further validation (Figure 4c). Notably, spermine, one of the natural polyamines present in eukaryotic cells, significantly induced cellular senescence and inhibited the proliferation of GM00038 cells during short‐term (Figure 4d,e; Figure S12, Supporting Information) and long‐term treatment (Figure 4f–i). A 14‐day treatment with spermine (1 µg mL^−1^) significantly increased the percentage of SA‐β‐gal positive cells and decreased the percentage of EdU‐positive cells. Therefore, upon a two‐step metabolites screening, spermine was identified as an aging‐promoting metabolite that significantly induced cellular senescence during long‐term treatment.

### 2.5 Validation of the Aging‐Promoting Effect of Spermine in Various Cell Types

**Figure 5:**
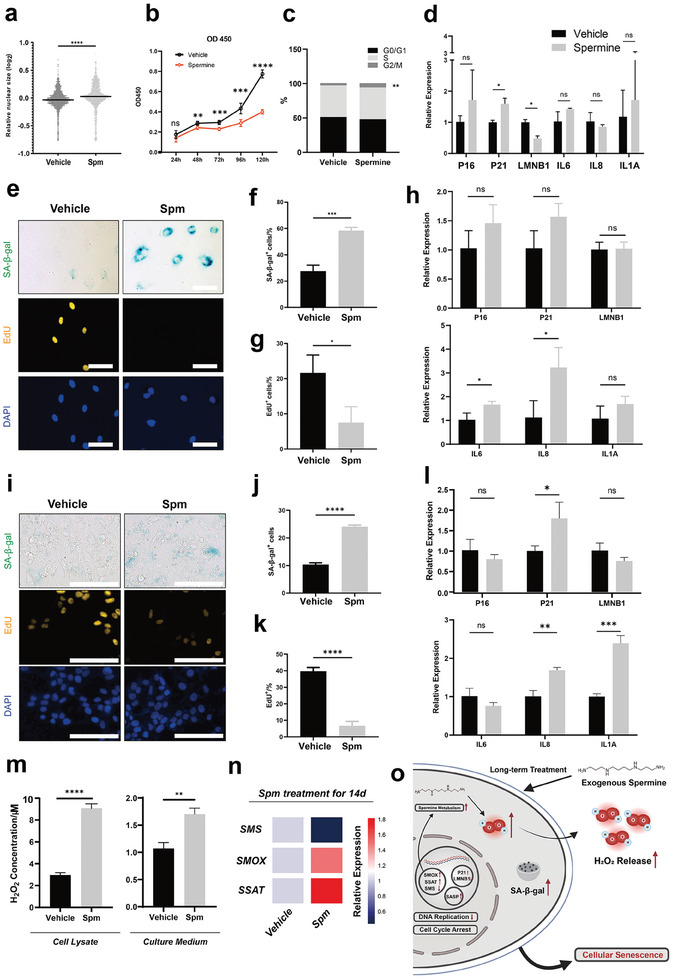
Spermine promotes cellular senescence in vitro. a) Quantification of the nuclear size of GM00038 cells treated with spermine for 14 days. Number of cells per group > 1500, cells from three biological replicates. b) The viability of GM00038 cells was examined by CCK8 after being treated with spermine (1 µg mL^−1^). n = 3 biological replicates. c) Cell cycle analysis of GM00038 cells treated with spermine (1 µg mL^−1^) for 7 days. d) mRNA levels of P16, P21, LMNB1, IL‐6, IL‐8, and IL‐1A in GM00038 cells treated with spermine (1 µg mL^−1^), normalized to GAPDH mRNA (n = 3 per group). e) Representative images of SA‐β‐gal (n = 3 per group/4 images per n), EdU (yellow; n = 3 per group/4 images per n), and DAPI labeled nuclei (blue) of HUVECs treated with spermine (2 µg mL^−1^) for 5 days. Scale bar, 100 µm. f,g) Quantification of SA‐β‐gal^+^ cells (f) and EdU^+^ cells (g) in (d). At least 100 cells per n were calculated. h) mRNA levels of P16, P21, LMNB1, IL‐6, IL‐8, and IL‐1A in HUVECs treated with spermine (2 µg mL^−1^), normalized to GAPDH mRNA. (n = 3 per group). i) Representative images of SA‐β‐gal (n = 3 per group/4 images per n), EdU (yellow; n = 3 per group/4 images per n), and DAPI labeled nuclei (blue) of WRL68 treated with spermine (2 µg mL^−1^) for 12 days. Scale bar, 100 µm. j,k) Quantification of SA‐β‐gal^+^ cells (j) and EdU^+^ cells (k) in (h). At least 800 cells per n were calculated. l) mRNA levels of P16, P21, LMNB1, IL‐6, IL‐8, and IL‐1A in WRL68 treated with spermine (2 µg mL^−1^) for 7 days, normalized to GAPDH mRNA. (n = 3 per group). m) H_2_O_2_ levels in GM00038 treated with spermine (1 µg mL^−1^) for 14 days (left, n = 3 per group). The amount of H_2_O_2_ released into the cell culture medium within 24 h after GM00038 cells were treated with spermine (1 µg mL^−1^) for 13 days (right, n = 3 per group). n) mRNA levels of SMS, SMOX and SSAT in GM00038 cells treated with spermine (1 µg mL^−1^) for 14 days, normalized to GAPDH mRNA (n = 5 per group). o) Exogenous Spermine treatment could induce typical cellular senescence phenotype and lead to increased overall H_2_O_2_ level in vitro. Data are means ± SD of biologically independent samples. Statistical significance was calculated using a two‐tailed *t*‐test (*p < 0.05; **p < 0.01; ***p < 0.001; ****p < 0.0001).

As spermine exhibited potential aging‐promoting function during long‐term treatment in GM00038 cells, we first evaluated whether this effect was dose‐dependent. Our results indicated that spermine could induce the aging‐promoting effect within a certain concentration range, whereas excessively high concentrations may greatly impair cell viability (Figure S13, Supporting Information). We then used effective concentrations to investigate the specific cellular and molecular response during treatment. The nuclear size of GM00038 cells was significantly elevated and more cells exhibited enlarged size and flattened shape after 14‐day spermine treatment (**Figure** **5**a; Figure S14, Supporting Information). Besides, spermine treatment caused the cell cycle arrest at the G_2_/M phase, as evidenced by decreased cell viability and increased percentage of G_2_/M phase cells (Figure 5c). Real‐time PCR analysis revealed significantly upregulated expression of P21 and downregulated expression of LMNB1, while P16 and senescence‐associated secretory phenotype (SASP)‐related genes, including IL‐8 and IL‐1A, also showed an increased expression (Figure 5d) after 14‐day spermine treatment in GM00038 cells. In summary, spermine treatment induced typical senescence phenotype accompanied by cell cycle arrest and activation of senescence‐related genes. In addition to skin fibroblasts that are developed from ectoderm, we wondered whether spermine could also induce cellular senescence in other cell types. Therefore, Human Umbilical Vein Endothelial Cells (HUVECs) developed from mesoderm and WRL‐68, normal human liver cells developed from endoderm,^[^ ^49^ ^]^ were applied to investigate the impact of spermine. After 5‐day spermine (2 µg mL^−1^) treatment, HUVECs exhibited typical senescence characteristics, increased SA‐β‐gal positive cells, and decreased EdU positive cells (Figure 5e–g). Real‐time PCR revealed significantly increased expression of IL‐6 and IL‐8 as well as up‐regulation of P16, P21, and IL‐1A gene expression (Figure 5h). Similarly, increased SA‐β‐gal positive cells and decreased EdU positive cells were also observed in WRL‐68 cells treated with spermine (2 µg mL^−1^) for 14 days (Figure 5i–k), and a 7‐day spermine treatment significantly increased the expression of P21, IL‐8 and IL‐1A (Figure 5l). The polyamine metabolism is accompanied by the generation of H_2_O_2_ and exogenous spermine treatment also induced significantly increased accumulation and release of H_2_O_2_ (Figure 5m). Real‐time PCR showed increased expression of spermine oxidase (SMO) and spermidine/spermine‐N^1^‐acetyltransferase (SSAT) while the expression of spermine synthase (SMS) was significantly inhibited in GM00038 cells treated with spermine (1 µg mL^−1^) (Figure 5n). Hence, Spermine treatment could induce typical cellular senescence in cells from different germ layers and lead to metabolic changes accompanied by increased H_2_O_2_ production in vitro (Figure 5o).

Polyamines, including putrescine, spermidine, and spermine, are basic organic products in all eukaryotes and have been proven to be essential for eukaryotic cell growth and survival.^[^ ^12^, ^50^ ^]^ Previous studies have identified PTEN‐PI3K‐mTOR complex 1, WNT signaling, and RAS pathway interplayed with polyamine metabolisms.^[^ ^51^ ^]^ We confirmed the senescence‐induction ability of spermine in vitro, indicating a potential interplay between polyamine metabolism and cellular senescence. We evaluated the expression of two essential polyamine metabolism‐related enzymes, spermidine synthase (SRM) and spermine synthase (SMS),^[^ ^52^ ^]^ in the young and aged cell lines used in single‐cell metabolic assay. Surprisingly, the expression of SRM and SMS in aged cells was significantly lower than that in young cells (Figure S15, Supporting Information). The decreased level of SRM and SMS indicated that spermine‐related metabolisms were less active in aged cells and this may partially explain the over‐accumulation of spermine in aged and senescent cells.^[^ ^53^ ^]^ To further verify the accumulation of spermine in aged cells, we performed a multi‐cellular metabolic assay using MS on young and aged fibroblasts. Considering that most of the SERS signals originate from the molecules adsorbed onto NPs with relatively high affinity, we utilized matrix‐assisted laser desorption (MALDI) for MS analysis to verify these molecules. Since Ag NPs have been recognized as an effective matrix for the MALDI MS analysis of small molecules, this method can be utilized to identify metabolites that are enriched on the surface of citrate‐reduced Ag colloids.^[^ ^54^ ^]^ Sample preparation followed the same method as the SERS preparation. The cell lysate containing metabolic small molecules and Ag NPs were mixed and the supernatant was then discarded after centrifugation. The Ag NPs with adsorbed metabolic molecules were then subjected to Fourier transform ion cyclotron resonance (FT‐ICR) MS (see details in Methods). The positive ion mode mass spectrum of a representative fibroblast cell line illustrates the strongest signal at 322.72 m z^−1^, representing the background (BG) signal of Ag NPs, along with several metabolite peaks (Figure S16a,b, Supporting Information). Notably, the peak corresponding to spermine in the positive ion mode appears at 225.20 m z^−1^. After normalization of the mass spectra of the samples and the BG, it can be found that the signal of spermine in the three aged cell lines (AG05247, AG06279 and AG09602) is significantly higher than that in the young cells (GM00038) (Figure S16c, Supporting Information). This finding is consistent with the results obtained from the SERS single‐cell assay, highlighting the accumulation of spermine in aged cells. In vitro supplementation of spermine may break the existing metabolic homeostasis and lead to cellular senescence. The increased H_2_O_2_ production after exogenous spermine treatment at least partially contributes to the gradual senescence. However, the specific mechanisms between spermine metabolism and cellular senescence in natural aging still need further investigation. In addition, polyamine concentrations significantly altered in many aging‐related diseases.^[^ ^55^ ^]^ Spermine level was elevated in brain tissues and polyamine‐related gene expression was significantly dysregulated in cortical surface tissue in Alzheimer's Disease.^[^ ^56^ ^]^ Increased levels of spermine and polyamines were also found in Parkinson's Disease.^[^ ^57^ ^]^ These results highlighted the importance of polyamine metabolisms in aging‐related diseases. Considering spermine was identified as an inducer of cellular senescence, modulation of spermine‐related metabolic pathways may also offer an opportunity to interfere with aging‐related diseases and create new treatment options.

Previous studies have demonstrated oral supplementation of spermine (3 mM) extends the lifespan of mice and exerts cardioprotective effects,^[^ ^58^ ^]^ which seems to be contrary to our conclusions. Considering the distinct metabolic characteristics between human and mouse cells, the differences in species may be responsible for the differences in results. It should be noticed that oral supplementation of certain metabolites undergoes digestive processes, and its influence on mice's digestive system and gut microbiome may greatly affect the overall metabolism and health. In addition, mice's intake of water is intermittent so the supplementation of spermine won't be similar to in vitro treatment as cells are always in a state of spermine addition. Direct spermine treatment on various cell types sets a model to investigate the impact of a single metabolite on cellular senescence. The potential connections between the rejuvenating effect at the systemic level and the aging‐promoting effect at the cellular level need more investigation, as do the unknown mechanisms involved in spermine intake. Thus, we summarized that spermine could indeed induce cellular senescence in various cell types in an in vitro supplementation examination.

## 3 Conclusion

In this study, we developed a label‐free single‐cell metabolic analysis system based on the SERS technique and the SlipChip microfluidic device. This system combines the fingerprint features of SERS spectral sets (i.e., SERSomes) and the multistep fluidic operation capability of SlipChip. We performed robust single‐cell analysis on various types of cancer cells and senescence‐based single‐cell analysis in young and aged fibroblasts, and the Raman peak at 1571 cm^−1^ was identified as the aging marker‐related band feature. Further, in a two‐step study, we screened for aging‐promoting metabolites based on 12 metabolites associated with the 1571 cm^−1^ characteristic peak. We identified spermine as an aging‐promoting metabolite that could induce typical cellular senescence phenotype during long‐term in vitro treatment. Spermine treatment induced G_2_/M phase cell cycle arrest and increased senescence‐ and SASP‐related gene expression. Notably, spermine induced cellular senescence in multiple cell types, including fibroblasts, HUVECs and WRL‐68 cells that are derived from all three different germ layers and exogenous spermine treatment significantly increased the accumulation and release of H_2_O_2_. Additionally, this platform can be further applied in biological research and clinical therapy, such as decoding cell metabolic heterogeneity,^[^ ^59^ ^]^ understanding metabolic remodeling of tumor microenvironment,^[^ ^60^ ^]^ and predicting response to therapy.^[^ ^61^ ^]^ The integration of the SlipChip‐SERS microfluidic platform with other analytical technologies, such as high‐throughput sequencing, is expected to enable multi‐omics analysis of single cells, reveal the molecular mechanisms of cellular metabolism and advancing comprehensive biology analysis. Future enhancements may involve improving the efficiency of using cell samples by designing specialized capture features on the SlipChip based on hydrodynamic^[^ ^62^ ^]^ or size‐exclusion principles,^[^ ^63^ ^]^ as well as integrating microscopes with image recognition algorithms to efficiently identify single cells and enhance detection throughput. With the refinement of the metabolite spectral database and spectral unmixing algorithms, coupled with the use of NPs with different surface properties (which have different affinities to specific metabolites) for detection, the molecular identification capability of SERS will be greatly improved. Furthermore, the SlipChip‐SERS platform can be combined with the digital colloid‐enhanced Raman spectroscopy (dCERS) technique^[^ ^29^ ^]^ for highly accurate quantification of specific metabolites.

## 4 Experimental Section

### Materials and Instrumentation

Citrate sodium (98%), silver nitrate (AgNO_3_) (AR, 99.8%), saponin (BR, 10–25%), tetradecane, silicone oil, and dichlorodimethylsilane were purchased from Aladdin (Shanghai, China). SDS was obtained from Beyotime (Shanghai, China). TritonX‐100 was obtained from Sangon Biotech (Shanghai, China). 20 pure metabolites were used for SERS measurement (see details in Table S1, Supporting Information). Sodium hydroxide, ceric ammonium nitrate, nitric acid, perchloric acid, ammonium fluoride, hydrofluoric acid, sulfuric acid, hydrogen peroxide, chloroform, acetone, and ethanol were obtained from SinoPharm Chemical Reagent (Beijing, China). Calcein acetoxymethyl ester (Calcein AM) (≥ 95%) was purchased from Yeasen Biotechnology (Shanghai, China). Quartz glass plates coated with chromium and photoresist gel layers were purchased from Shaoguang Global Blankmask Technology (Changsha, China). K_3_Fe(CN)_6_, K_4_Fe(CN)_6_·3H_2_O, MgCl_2_·6H_2_O, citric acid monohydrate, N,N‐dimethylformamide, CuSO_4_·5H_2_O and L‐sodium ascorbate were purchased from Sigma–Aldrich. NaCl and Na_2_HPO_4_ were obtained from Sinopharm Chemical Reagent (China). Sulfo‐cyanine‐3‐azide was purchased from Lumiprobe (United States). Ultrapure water (18.2 MΩ) was used for all experiments.

UV–vis spectra of the NPs were collected from a UV1900 UV–vis spectrophotometer (Aucybest, Shanghai, China). The hydrodynamic diameters of the NPs were characterized by a Zetasizer Nano ZSP (Malvern, UK). The morphology of the NPs was obtained from a JEM‐2100F transmission electron microscope (JEOL, Tokyo, Japan). All SERS spectra were acquired by a confocal Raman system (XploRA INV, Horiba). Fluorescence images were captured by an inverted microscope (Eclipse Ti2, Nikon). The mass spectrometry was captured by a Fourier transform ion cyclotron resonance mass spectrometer (FT‐ICRMS, SolariX 7.0T, Bruker, United States).

### Design and Preparation of the SlipChip

The SlipChip was designed by AutoCAD (San Rafael, CA, USA) (Figure S1a, Supporting Information) and consists of two plates made of quartz, one on top and one on bottom. The bottom plates were imprinted with microwell arrays, which contained 768 microwells. The geometry of the microwells was designed to be 260 µm in diameter and 30 µm in depth, which was capable of generating droplets of 1.4 nL. The center‐to‐center distance of microwells is 475 µm. The top plate contains loading channels and large receiving microwells. The depth of the loading channel was designed to be 40 µm, and the width was 340 µm. The receiving microwells were designed to be 320 µm in diameter and 40 µm in depth, which were capable of generating droplets of 2.9 nL.

The fabrication was based on a photolithography method and a wet‐etching protocol described previously.^[^ ^64^ ^]^ The etching microstructure was monitored using a profilometer (Brucker, Billerica, MA, USA) to reach the designed size. The surface of the chip was washed with piranha solution (H_2_SO_4_:H_2_O_2_ = 2:1 v/v) and plasma before each use. Then it was hydrophobized based on a previously described protocol.^[^ ^65^ ^]^

### Cell Culture

Human cervical cancer (HeLa) cells and human breast cancer (MCF‐7) cells were obtained from the Shanghai Institute for Biological Sciences (Shanghai, China). Cells were cultured in Dulbecco's Modified Eagle Medium (DMEM, Meilunbio, China) supplemented with 10% fetal bovine serum (FBS, KEL biotech, United States) and 1% penicillin–streptomycin (Gibco, United States) at 37 °C with 5% CO_2_.

Young human fibroblasts (GM00038, 9 YR) and aged fibroblasts (cat. No. AG09602, 92 YR; AG05247, 87 YR; AG06279, 81 YR) were obtained from Coriell Institute for Medical Research (United States). Cells were cultured in DMEM supplemented with 15% FBS and 1% penicillin–streptomycin at 37 °C with 5% CO_2_.

Human umbilical vein endothelial cells (CRL‐1730) were obtained from ATCC (United States). Cells were cultured in F‐12K (ATCC, United States) supplemented with 10% FBS, 1% endothelial cell growth supplement (Sciencell, United States), 100 µg mL^−1^ heparin (Macklin, China) and 1% penicillin–streptomycin at 37 °C with 5% CO_2_. Human normal liver cells (WRL‐68) were obtained from ATCC (United States). Cells were cultured in DMEM supplemented with 10% FBS and 1% penicillin–streptomycin at 37 °C with 5% CO_2_.

### Calcein AM Staining

Cells were digested and washed twice with PBS to completely remove residual esterase, followed by the addition of 5 µM staining solution to stain the cells and incubation for 25 min at 37 °C. The cells were washed twice with PBS and resuspended in PBS. 15 µL of the cell suspension was injected into the SlipChip and inspected by fluorescence microscope (laser: 485 nm, objective lens: 20×).

### Preparation of Ag NPs

The preparation of citrate‐stabilized Ag NPs was carried out with a slight modification of the Lee–Meisel method.^[^ ^66^ ^]^ Initially, AgNO_3_ (12.3 mg) was dissolved in 100 mL of ultrapure water. This solution was then brought to a boil under constant stirring. Subsequently, 2 mL of a 1% sodium citrate solution was quickly added. The boiling was maintained for 1 h, after which the mixture was allowed to cool to room temperature while stirring continued. The colloidal solution was then stored at 4 °C. The theoretical concentration of the Ag NPs in this preparation was approximately 0.2 nM. For concentration purposes, 1 mL of the Ag colloid was centrifuged at 5000 rpm for 8 min. After centrifugation, 950 µL of the supernatant was discarded, resulting in a final concentration of 4 nM.

### Preparation of Cell Lysates

Several common cell membrane‐breaking agents were formulated using PBS as the solvent with an initial concentration of 2% TritonX‐100, 2% SDS, and 0.4% saponin. 50 µL of detergent was taken and added 50 µL of 4 nM Ag colloid to mix well to obtain a mixture, which was added to cells for rupturing the membrane according to the volume ratio of 1:1.

### Operation of the SlipChip

The SlipChip was assembled into an oil phase composed of tetradecane and silicone oil (tetradecane: silicone oil = 1:1 v/v). The cell suspension was loaded into the SlipChip in the first step. Then the transporting microwell containing a single cell on the bottom chip can slip relative to overlap with the large receiving wells on the top chip to form the first series of droplets. Subsequently, the bottom plate can slip relative to the top plate to another loading channel, and the second solution containing the Ag NPs and saponin can be introduced into the device. Lastly, slip again and merge the transporting wells on the bottom with the receiving wells on the top chip to form a second series of droplets (Figure 1a). After the sample loading was completed, the chip was placed at room temperature for 60 min to ensure that the single cell and particles are fully mixed and metabolites are released from the cell.

### SERS Measurements

For testing of droplets in SlipChip, SERS mapping of single‐cell droplets was performed on a confocal Raman system at 6 × 6 (step size: 10 µm) through a 10× objective lens at a power of 12.7 mW, with an integration time of 1 s per spectrum.

For in‐tube testing, cell lysates/pure metabolites dissolved in ultrapure water (10 mM) were mixed with Ag NPs at a volume ratio of 1:1. 10 µL of the mixture was injected into a quartz capillary (ID: 1 mm, OD: 2 mm) for SERS measurements on a confocal Raman system. Using a 638 nm incident laser (power: 12.7 mW), each spectrum was acquired through a 10× objective lens for 1 s.

### Metabolites Treatment

Metabolite candidates were dissolved in either water or DMSO to make stock solutions. Spermine (1 µg mL^−1^), taurine (20 µg mL^−1^), L‐glutamine (50 µg mL^−1^), vitamin B6 (25 µg mL^−1^), acetylcholine chloride (20 µg mL^−1^), cystine (10 µg mL^−1^), L‐carnosine (50 µg mL^−1^), isoleucine (10 µg mL^−1^), xanthine (2 µg mL^−1^), Serotonin (4 µg mL^−1^), hypoxanthine (10 µg mL^−1^) and adenine (10 µg mL^−1^) were added into GM00038 culture medium for 7 or 14 days. Spermine (2 µg mL^−1^) was added into HUVEC or WRL‐68 medium for specific time‐point evaluation.

### FACS‐Based SA‐β‐Gal Detection

SA‐β‐gal intensity evaluated by FACS was performed as previously described.^[^ ^67^ ^]^ Briefly, cells were incubated with fresh medium containing 100 nM Bafilomycin A1 (MCE, United States) for 1 h, and 33.3 µM C12FDG (MCE) was added into the medium for a further 3 h‐incubation. Cells were then washed and trypsinized. After being washed 3 times with PBS (Meilunbio), the cells were acquired on BD FACS Aria II. MFI was quantified by FlowJo.

### SA‐β‐Gal and EdU Staining

SA‐β‐gal and EdU staining was performed as previously described.^[^ ^68^ ^]^ Briefly, cells were plated on coverslips in 24‐well plates and incubated with 10 µM EdU (Lumiprobe) at 37 °C with 5% CO_2_ for 1–16 h according to the cell doubling time. Cells were fixed with 2% (wt/vol) formaldehyde + 0.2% (wt/vol) glutaraldehyde (Sigma–Aldrich) and stained with SA‐β‐gal staining solution prepared freshly for 12–16 h. The staining solution was removed and cells were fixed with 4% (wt/vol) formaldehyde (Sigma–Aldrich). Cells were then incubated with 100 mM Tris (Shanghai Yuanye Bio‐Techonology) and permeabilized with 0.1% TritonX100 (Beyotime, China). EdU staining solution was prepared freshly and added to the sample for 30 min in the dark at RT. Cells were then incubated with 2 µg mL^−1^ DAPI (Cell Signaling Technology) in the dark at RT for 20 min. Coverslips were mounted and dried overnight. Images were captured by Leica DM6 B and quantified by ImageJ.

### Real‐Time PCR Analysis

For RNA extraction, cells were incubated with RNAiso Plus (Takara, Japan) at RT for 10 min and total RNA was reversed transcribed using PrimeScript RT reagent Kit (Perfect Real Time) (Takara) according to the manufacturer's instructions. Real‐time PCR was performed using—TB Green Premix Ex Taq GC (Perfect Real Time) (Takara) according to the manufacturer's instructions and the reaction was performed on an ABI 7900HT Fast Real‐Time PCR System. Real‐time PCR results were analyzed by Expression suite v1.1 and relative expression was calculated using 2^−ΔΔCT^ method. Primer sets for human genes are listed in Table S2 (Supporting Information).

### CCK8 Analysis

The viability of GM00038 cells was evaluated with a CCK8 (Share‐bio, China) assay. Briefly, cells were plated in a 96‐well plate and after specific time points, cells were washed with PBS and CCK8 solution (10 µL in 100 µL medium) in the fresh medium was added to wells and incubated for 3 h at 37 °C with 5% CO_2_. OD450 was measured by a microplate reader. The cell growth curve is drawn according to the measured OD value.

### Cell Cycle Analysis

For cell cycle analysis, adhesion cells were trypsinized and suspended in PBS. Ice‐cold anhydrous ethanol was added into the cell suspension drop by drop accompanied with gently vortex to avoid cell aggregation. Cells were fixed at 4 °C overnight and washed by PBS for 3 times. Cell pellet was resuspended in DAPI staining solution (0.1% Trion X‐100 and 1 µg mL^−1^ DAPI in PBS) and incubated in the dark at RT for 10 min. Sample was then analyzed with flow cytometry. Cell cycle distribution was determined by FlowJo.

### H_2_O_2_ Measurement

The H_2_O_2_ level in cell lysate and cell culture supernatant were measured using Hydrogen Peroxide Assay Kit (Beyotime, China) according to the manufacturer's instructions.

### Mass Spectrometry Preparation and Detection

MALDI‐sourced multi‐cellular samples were used for FT‐ICRMS detection. The cell lysates were centrifuged at 14 000 rpm for 10 min to remove cellular debris, and 50 µL of supernatant containing metabolic small molecules was taken and added to 50 µL of 4 nM Ag colloid and mixed well to allow the particles to fully adsorb the metabolic molecules. The mixture was centrifuged at 5000 g for 10 min, the supernatant was discarded, and 10 µL of ultrapure water was added to resuspend the particles. The control group was Ag colloid without adsorbed metabolic molecules. Three biological replicates were prepared for each sample.

The droplets containing the particles were added to the test substrate of FT‐ICRMS and left to dry on the machine. After successful calibration of the instrument, 25% power was selected for excitation, and the acquisition interval was from 60 to 1500 m z^−1^. Five mass spectra were acquired for each droplet (including five in positive ion mode and five in negative ion mode). The data were analyzed by Bruker Data Analysis software.

### Data Processing and Analysis

We pre‐processed all spectral data and calculated spectral number using MATLAB R2022b. The spectra were smoothed using a Savitzky–Golay (SG) filter with a polynomial order of 3 and a window size of 5. The baseline removal was fitted to the baseline of each spectrum using adaptive iterative reweighted penalized least squares (airPLS) with an order of 3 and a lambda value of 10^7^. To calculate how many spectra to acquire in order to obtain statistically stable spectral information, the Pearson correlation coefficient (PCC) curve for one spectral set was calculated by randomly selecting two subsets in the spectral set and calculating the PCC values for the two averaged spectra.^[^ ^25^ ^]^

Spectral data were further analyzed using Python 3.10.9 and Random Forest was used for binary classification, and tenfold cross validation was used to prove the stability of the results by repeating it 10 times. The discriminating Raman shifts for two classes were screened out as classification features (contribution > 0.1%). Using these selected features, UMAP was used for data dimensionality reduction to visualize the separation of data. Hierarchical clustering was used to cluster the features, “ward” and “euclidean” were used as method and metric. Metabolic profiles were plotted on Pearson correlation heatmaps using “Hmisc” and “ggplot2” from the R package with a threshold of “ρ > 0.8, P < 0.05”.

### Statistical Analysis

The data are presented as mean ± SD. The normality of data distribution in each set of experiments was assessed using either the D'Agostino–Pearson omnibus test or the Shapiro–Wilk test. Statistical analyses were conducted using Prism v.9.0 (GraphPad). To assess statistical significance, the two‐tailed *t*‐test was applied for the comparison of the two groups and one‐way ANOVA was performed for comparisons of data with more than two groups. The statistical significance indicated in the figures was assigned as not significant (ns) *p* > 0.05; *p < 0.05; **p < 0.01; ***p < 0.001; ****p <0.0001.

## Conflict of Interest

The authors declare no conflict of interest.

## Author Contributions

J.Y. proposed the idea and designed the experiment. F.G.L., Q.J.L., and Y.L. performed the experiments, analyzed the data, and finished the original draft. S.Y.W. and H.R.C. assisted with the data processing. X.L. and Z.W.L. assisted with the experiments. F.S., F.F.Z., and J.Y. supervised the work, revised the manuscript, and gave reasonable proposals.

## Supporting information

Supporting Information

## Data Availability

The data that support the findings of this study are available from the corresponding author upon reasonable request.
