# Supplementary material for: A Single‐Cell Metabolic Profiling Characterizes Human Aging via SlipChip‐SERS
Source: Adv Sci (Weinh). 2024 Sep 4;11(41):2406668. doi: 10.1002/advs.202406668 (PMC11538647; doi:10.1002/advs.202406668)
Supplement: Supplementary file 1 — Supporting Information [file ADVS-11-2406668-s001.docx]

**Supporting Information for**

A Single-Cell Metabolic Profiling Characterizes Human Aging via SlipChip-SERS

Fugang Liu^aΔ^, Jiaqing Liu^aΔ^, Yang Luo^aΔ^, Siyi Wu^a^, Xu Liu^a^, Haoran Chen^a^, Zhewen Luo^a^, Haitao Yuan^a^, Feng Shen^a*^, Fangfang Zhu^a*^, and Jian Ye^a,b,c,d*^

^a^School of Biomedical Engineering, Shanghai Jiao Tong University, Shanghai 200030, China

^b^State Key Laboratory of Systems Medicine for Cancer, Shanghai Cancer Institute, Ren Ji Hospital, School of Medicine, Shanghai Jiao Tong University, Shanghai 200032, China

^c^Institute of Medical Robotics, Shanghai Jiao Tong University, Shanghai 200240, China

^d^Shanghai Key Laboratory of Gynecologic Oncology, Ren Ji Hospital, School of Medicine, Shanghai Jiao Tong University, Shanghai 200127, China

^Δ^These authors contributed equally to this work.

*To whom correspondence should be addressed: yejian78@sjtu.edu.cn (J. Y.); zhuff@sjtu.edu.cn (F. Z.); feng.shen@sjtu.edu.cn (F. S.)

**This PDF file includes:**

Figures S1 to S17

Tables S1 to S2


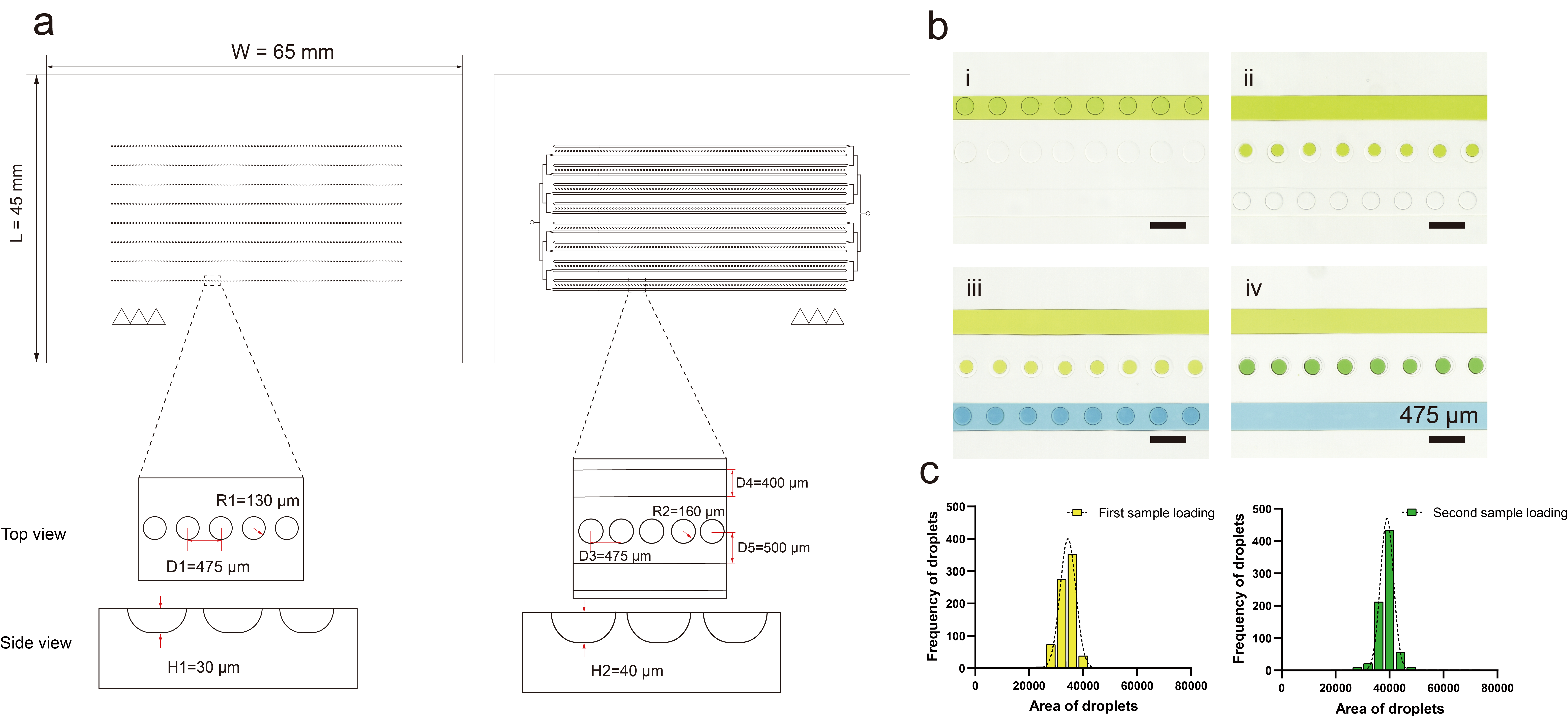


**Fig. S1.** Demonstration of the structure and function of the SlipChip. **a** The design of the SlipChip. **b** The bright-field image of the SlipChip illustrates the results after injecting food dye into the microchannel **i**, forming yellow dye droplets **ii**, injecting blue dye **iii**, and mixing blue and yellow dye droplets **iv**. **c** Statistical diagram of the droplet areas formed in steps **ii** and **iv**.


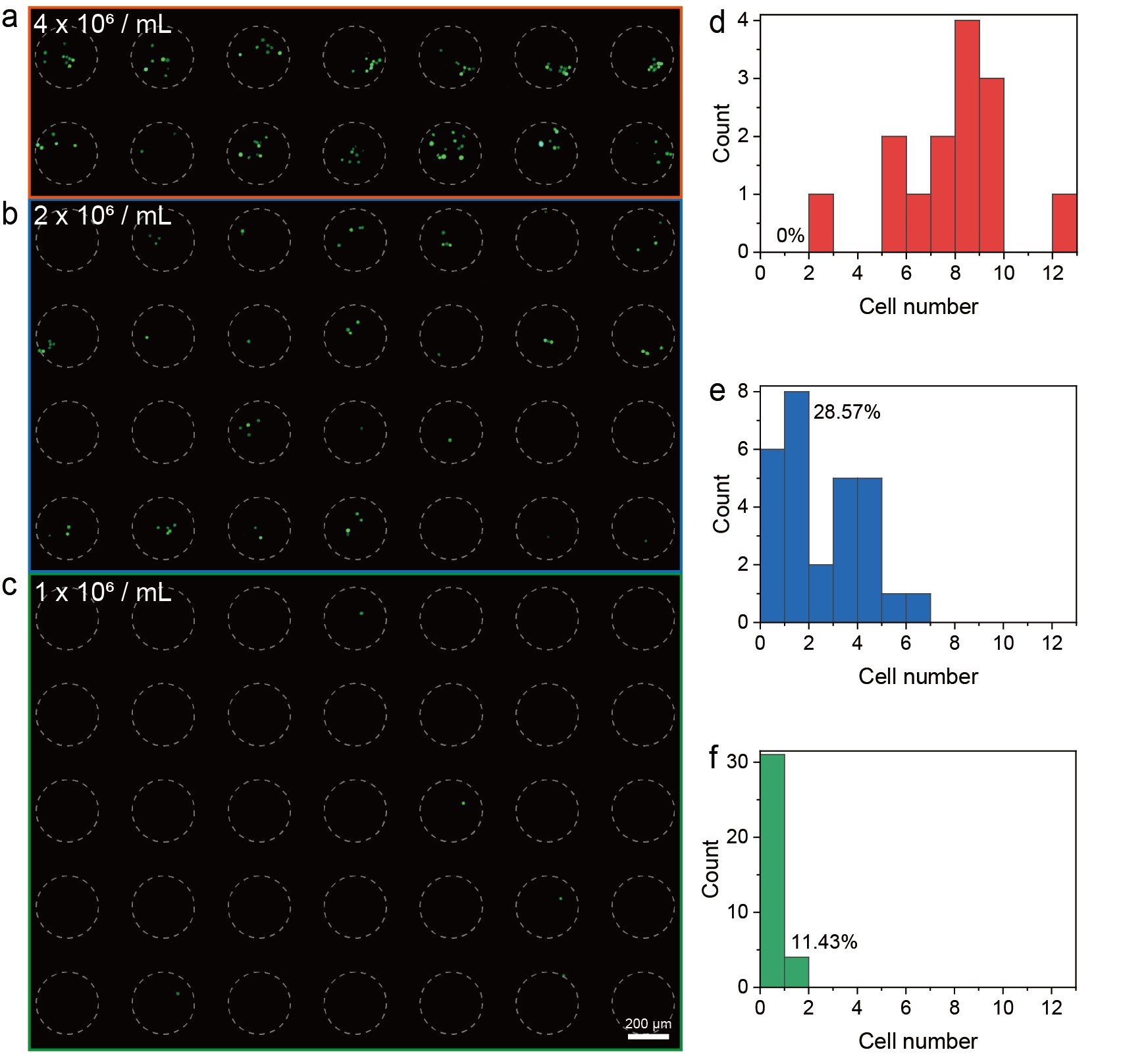


**Fig. S2.** Cell loading concentration influences the formation of single cell encapsulation events. **a-b** Fluorescence imaging of MCF-7 cells in microwells at different injected cell concentrations (4, 2, 1 × 10^6^ cells / mL). Cells are stained by Calcein AM. **d-f** Distribution of the number of cells per microwell in panels **a-c**, percentages show the frequency of occurrence of single cell wells.


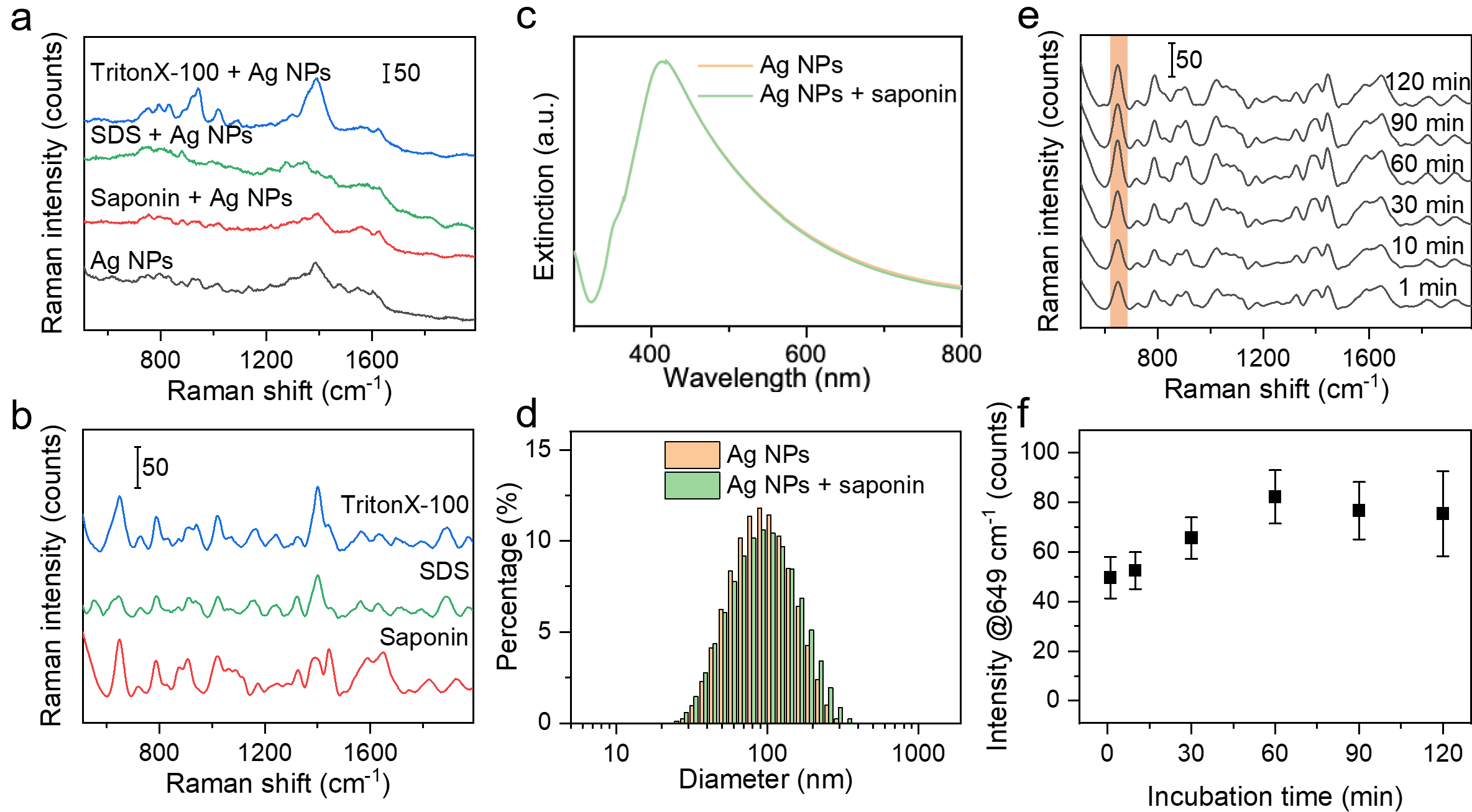


**Fig. S3.** SERS spectra of metabolites produced by cells treated with different detergents or times. **a** Averaged SERS spectra of the background by mixing Ag NPs with different detergents. **b** Averaged SERS spectra of MCF-7 cells incubated with different detergents mixing with Ag NPs for 1 h. **c** UV/Vis spectra, and **d** hydrodynamic diameter characterization of Ag NPs before (orange) and after (green) mixing with saponin. **e** Averaged SERS spectra of MCF-7 cells incubated with saponin mixing with Ag NPs for different times. **f** Variation of the intensity of SERS peak at 649 cm^-1^ with time.

**
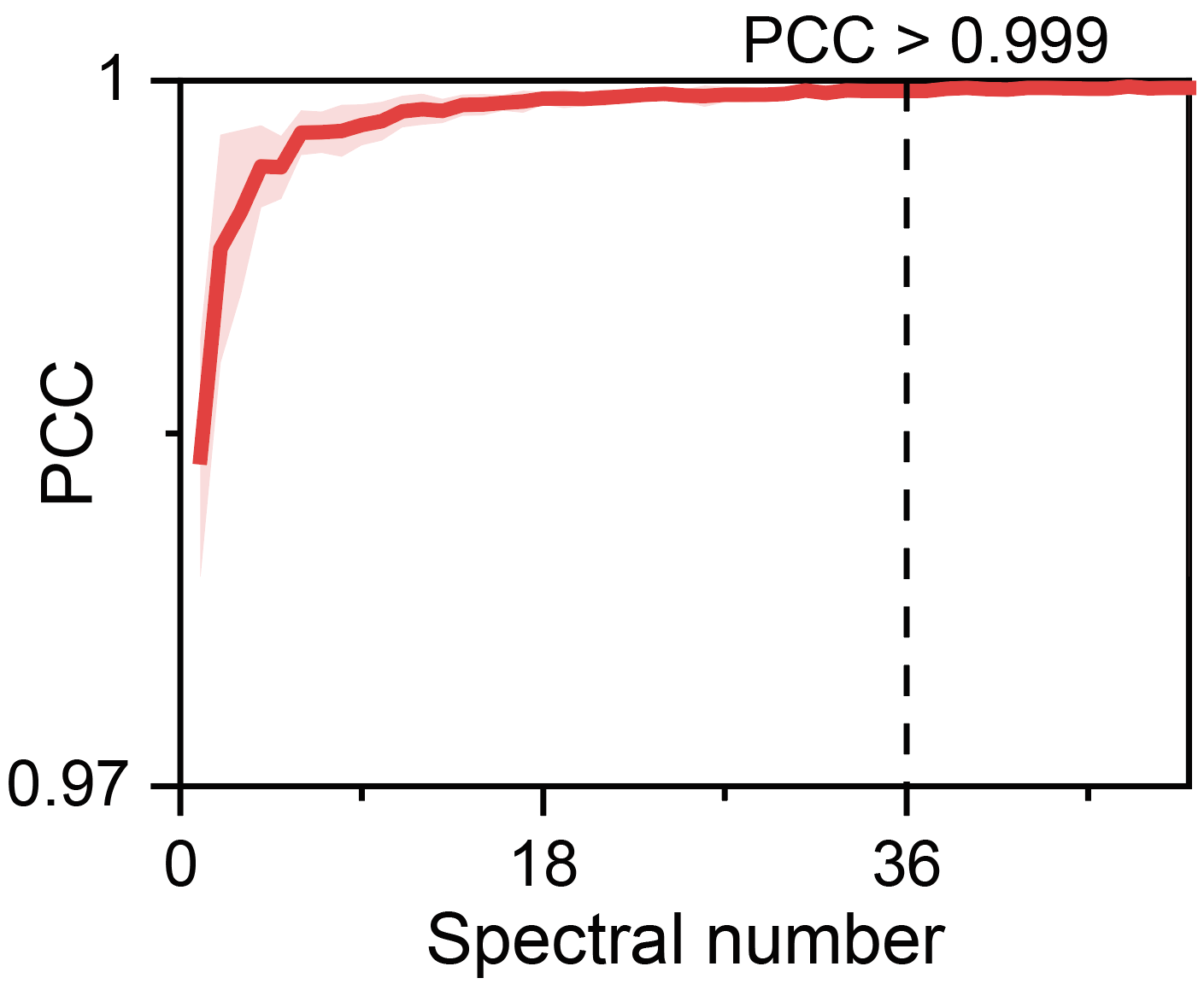
**

**Fig. S4.** The relationship between the average Pearson correlation coefficient (PCC) of 4 MCF-7 single-cells and 4 HeLa single-cells with the number of collected spectra.


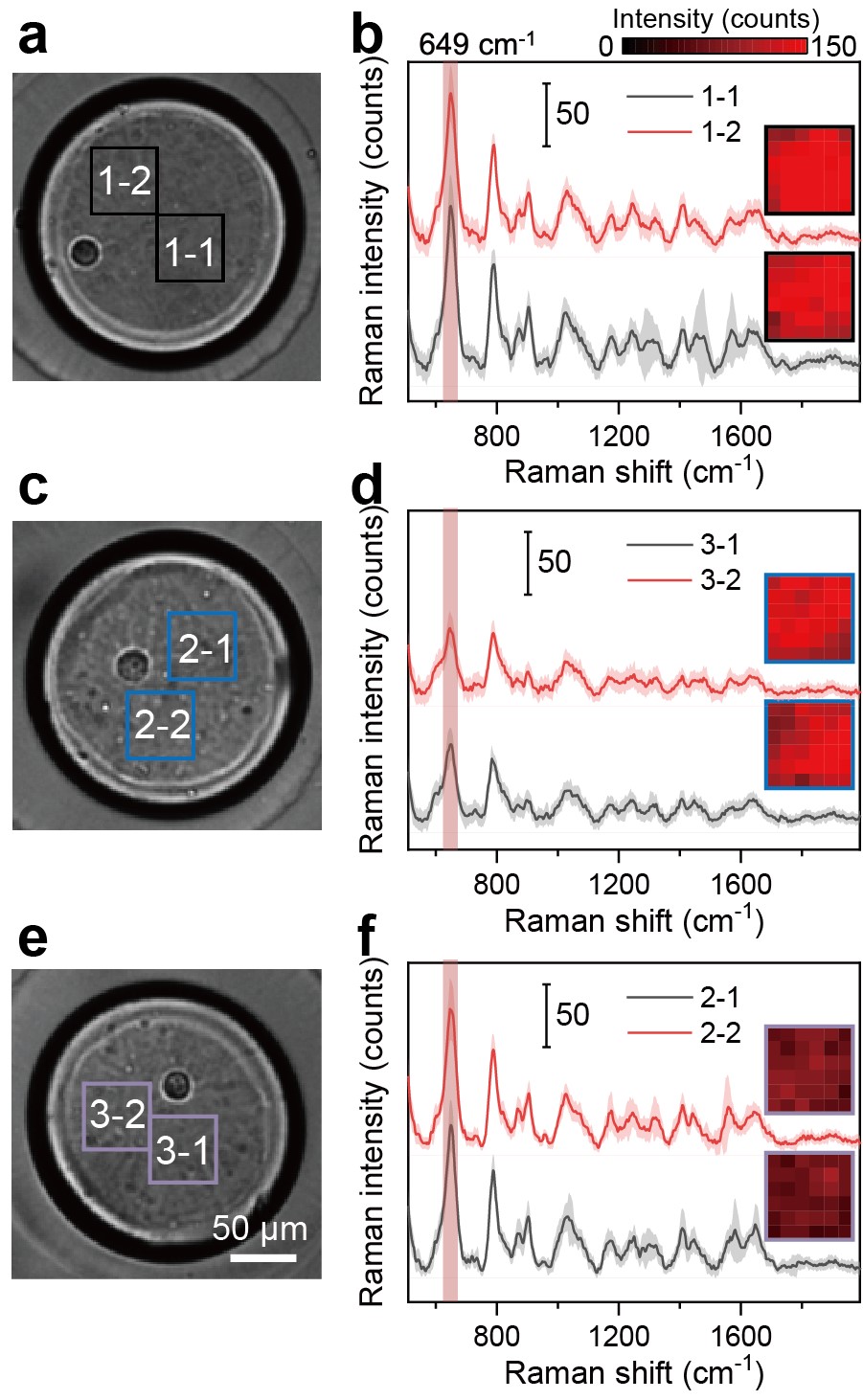


**Fig. S5.** Stability test of Raman signals in different regions of the same droplet. **a**, **c**, and **e** are the brightfield images of three single-cell droplets, and the boxes showed SERS test regions. **b**, **d**, and **f** showed the averaged spectra of the test regions and the reproducibility comparison of the peak at 649 cm^-1^ heatmap.


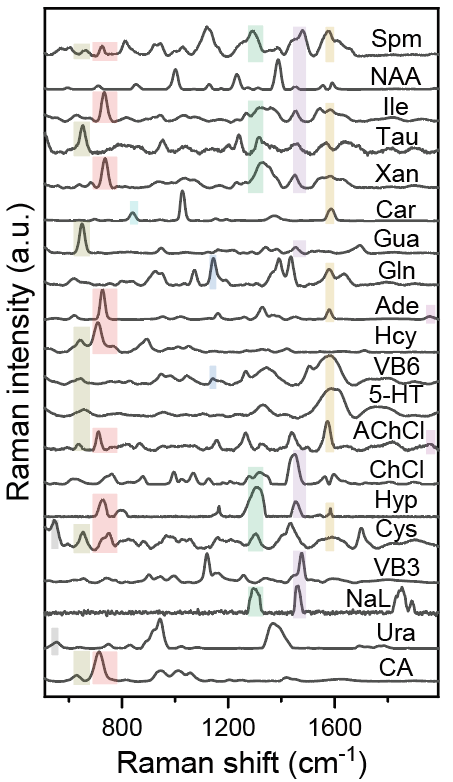


**Fig. S6.** Averaged SERS spectra of some potential assigned metabolites sharing high correlation Raman bands marked by shaded areas (see the abbreviations of all metabolites in Supplementary Table 1, 100 spectra were collected in for each metabolite).

**
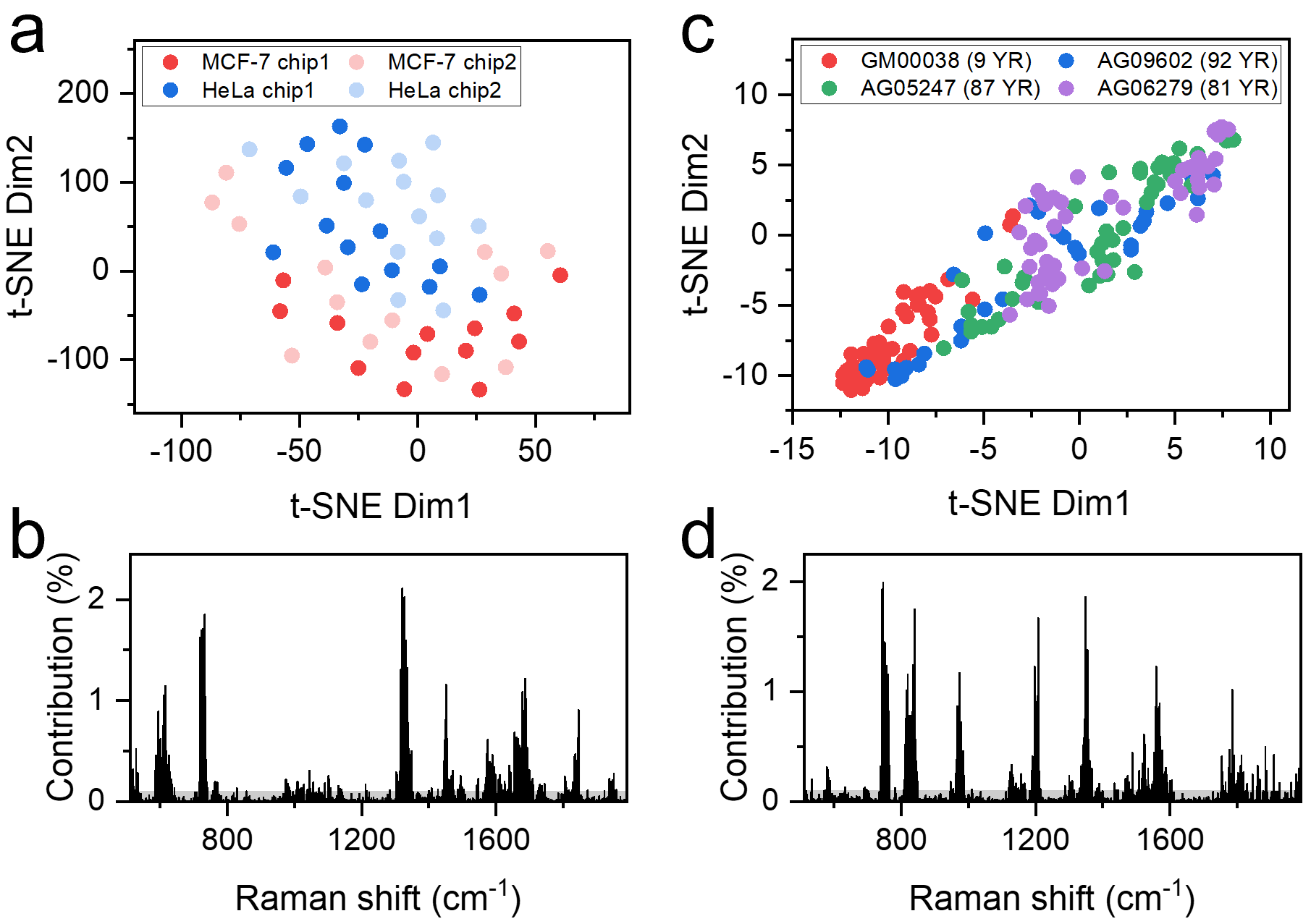
**

**Fig. S7.** Random forests based on the average spectra of single cells were screened for Raman shifts that discriminate between MCF-7 and HeLa cell lines (contribute > 0.1%).


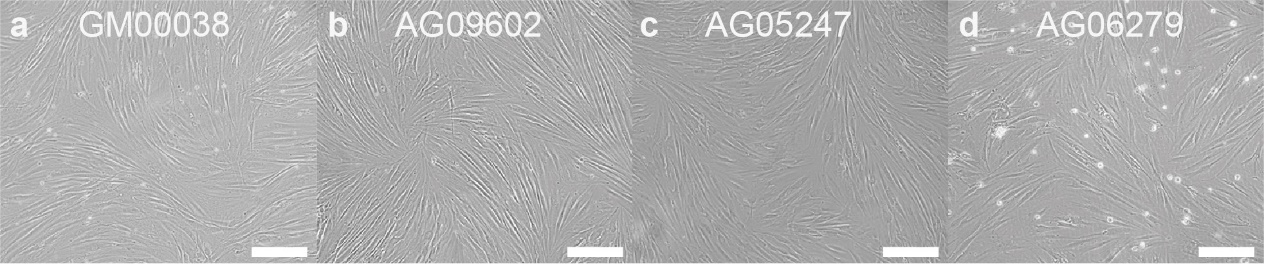


**Fig. S8.** Bright-field images of four types of fibroblast cells with different ages. All scale bars are 200 μm.

**
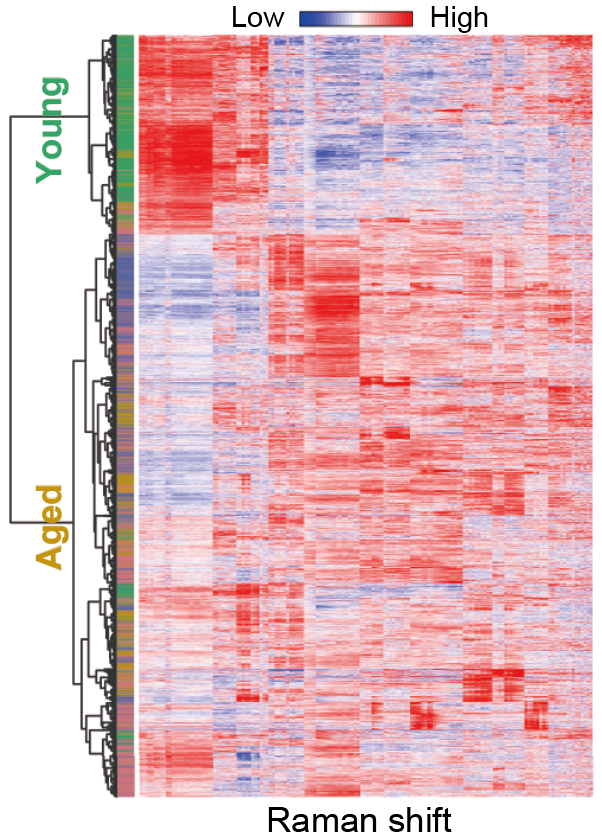
**

**Fig. S9.** Hierarchical clustering heatmap of the discriminating Raman shift profiles of young and aged cells. The color map shows the relative intensity with Raman shifts.

**
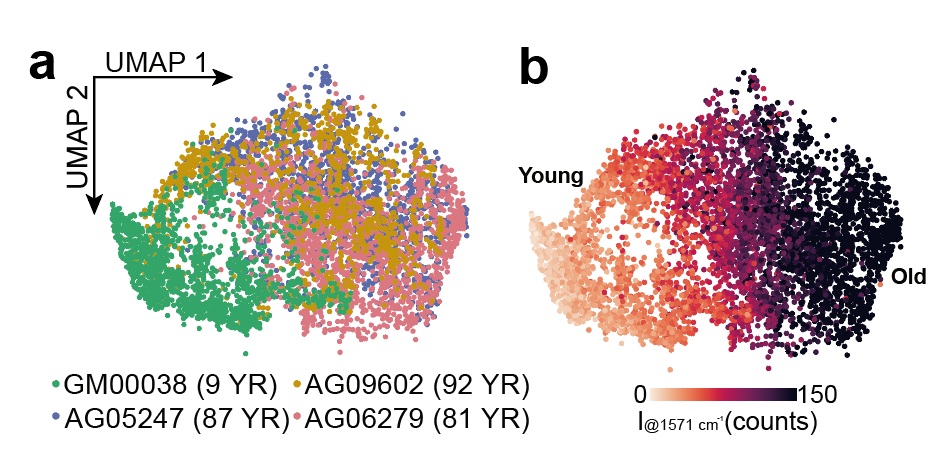
**

**Fig. S10.** **a** UMAP plots of four types of samples: GM00038 (green), AG09602 (yellow), AG05247 (purple), and AG06279 (pink). **b** All spectra are colored by the SERS intensity of 1571 cm^-1^ in UMAP plots.


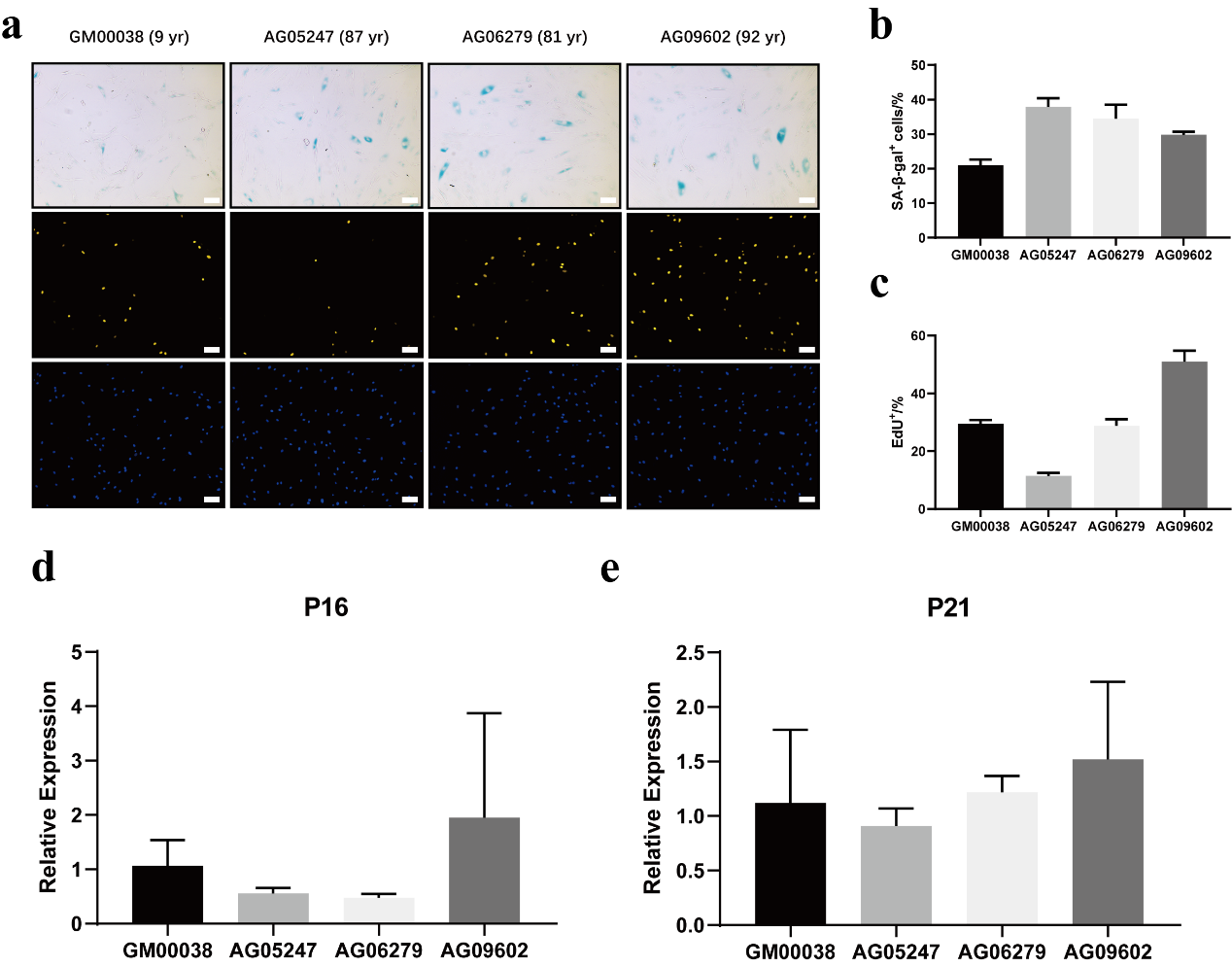


**Fig. S11. a** Representative SA-β-gal (n=3 per group/4 images per n), EdU (yellow; n=3 per group/4 images per n), and DAPI-labeled nuclei (blue) of four types of fibroblasts with different ages. Scale bar, 100μm. **b-c** Quantification of SA-β-gal+ cells **b** and EdU+ cells **c** in **a**. At least 300 cells per n were calculated. **d-e** mRNA levels of P16 **(d)** and P21 **e** in fibroblasts with different ages, normalized to GAPDH mRNA. (n=3 per group). Data are means ± SD of biologically independent samples.


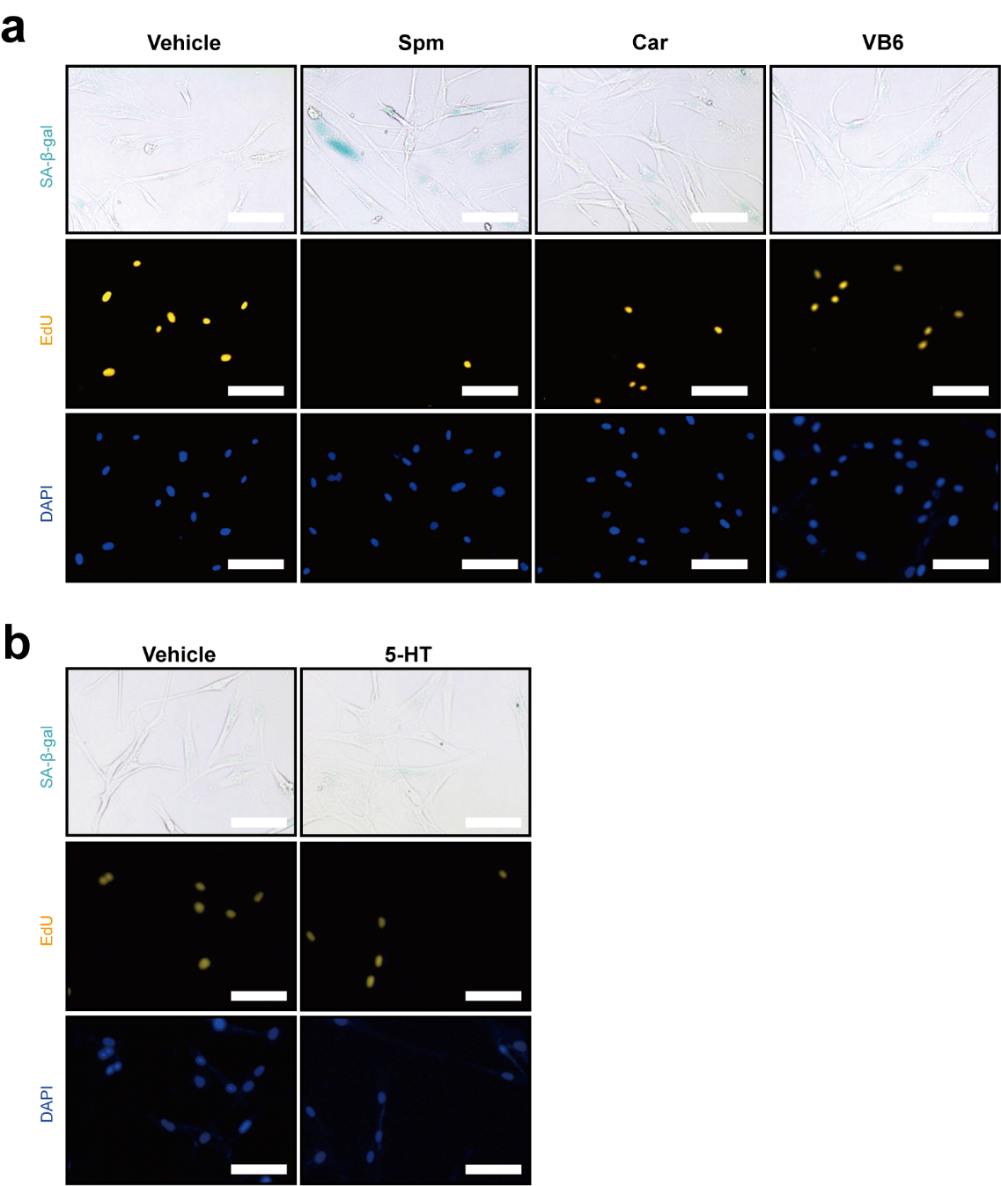


**Fig. S12.** Representative SA-β-gal (n=3 per group/4 images per n), EdU (yellow; n=3 per group/4 images per n), and DAPI-labeled nuclei (blue) of GM00038 cells in 7-day candidates screening. Water as solvent group **a** and DMSO as solvent group **b**. Scale bar, 100μm.


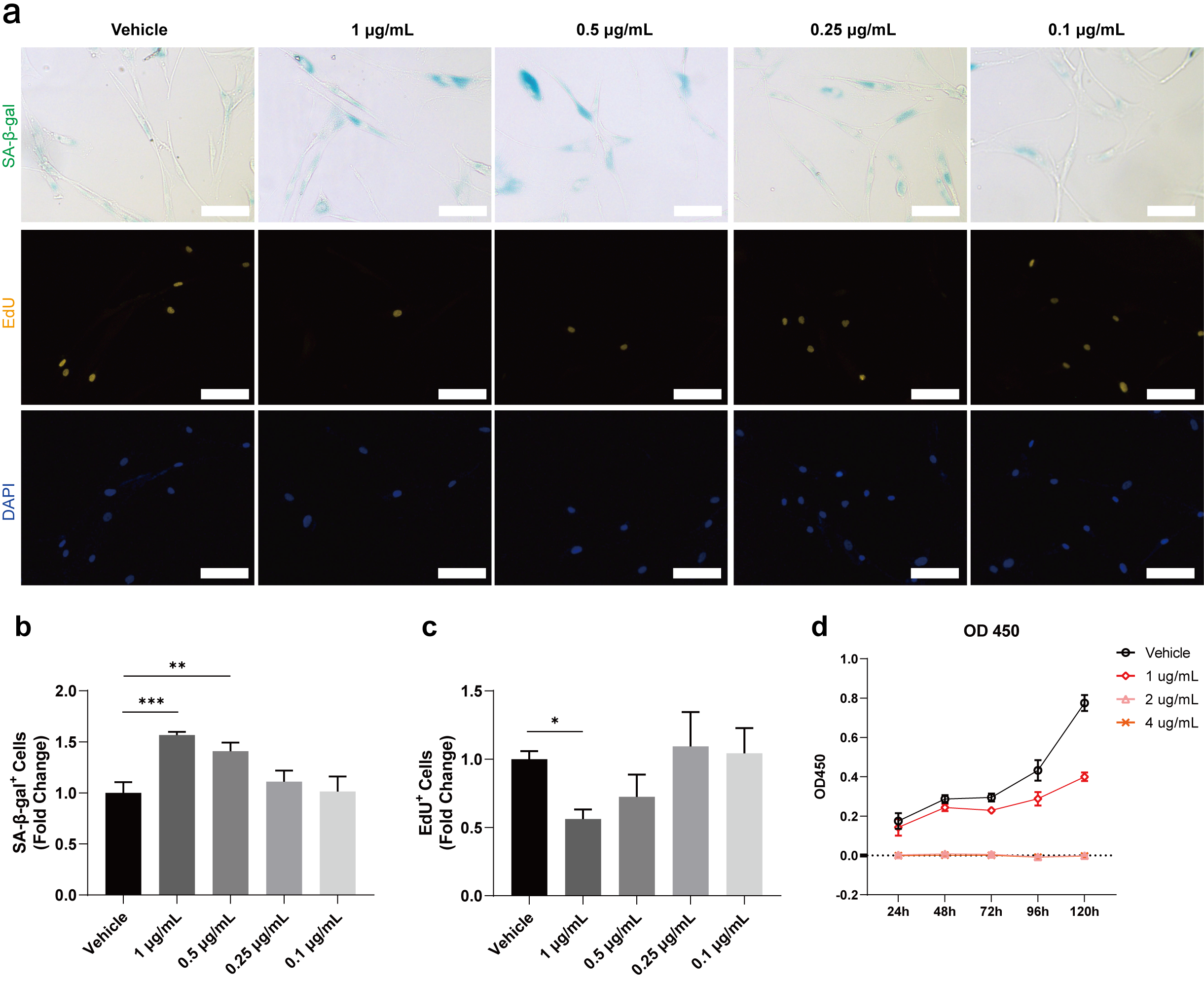
**Fig. S13.** The dose-dependent senescence induction ability of spermine *in vitro*. **a** Representative SA-β-gal (n=3 per group/6 images per n), EdU (yellow; n = 3 per group/6 images per n), and DAPI labeled nuclei (blue) images of GM00038 treated with different concentrations of spermine for 4 days. **b-c** Quantification of SA-β-gal^+^ cells and EdU^+^ cells in **a**. At least 150 cells per n were calculated (Normalized to Vehicle). **d** The viability of GM00038 cells was examined by CCK8 after being treated with relatively high concentrations of spermine. n = 3 biological replicates. Scale bar, 100 μm.

**
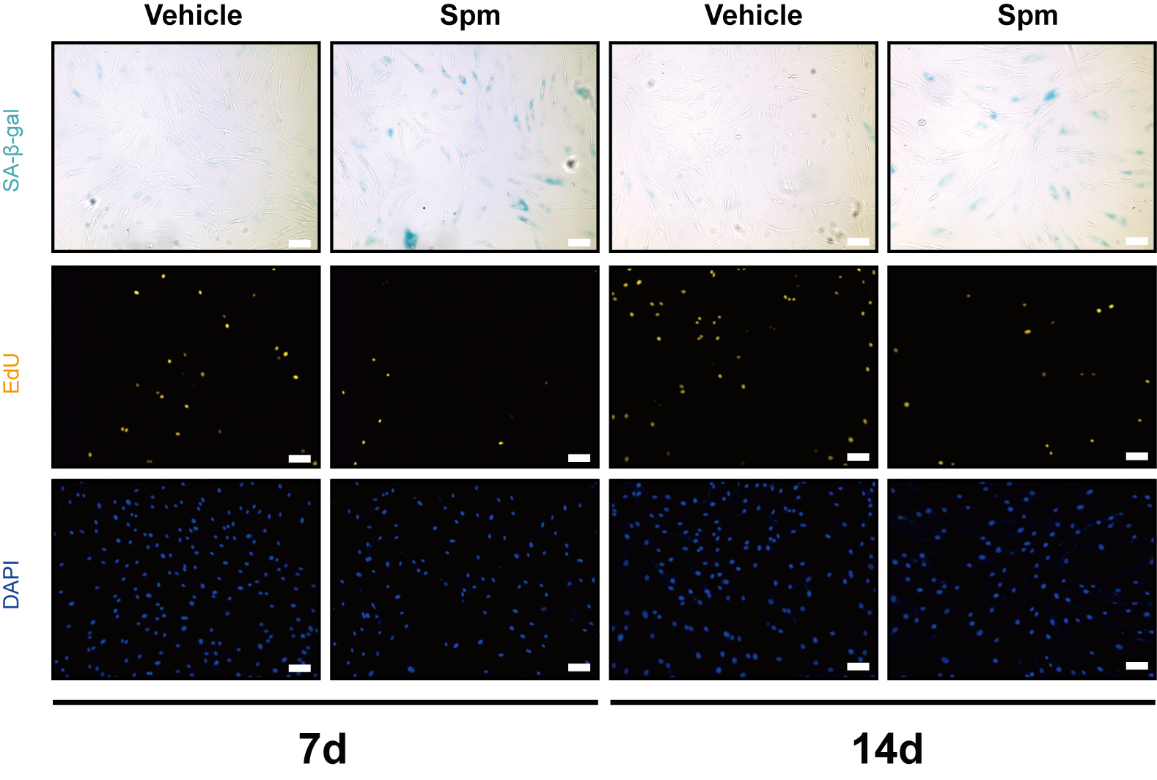
**

**Fig. S14.** Representative SA-β-gal (n=3 per group/4 images per n), EdU (yellow; n=3 per group/4 images per n), and DAPI-labeled nuclei (blue) of GM00038 cells treated with spermine (1 μg/mL). Scale bar, 100μm.

**
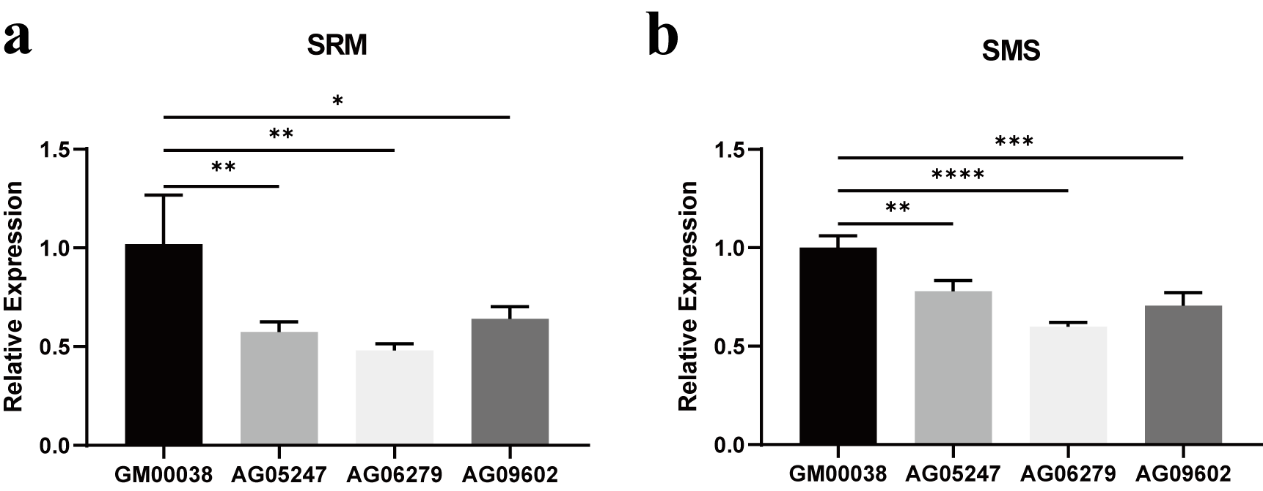
**

**Fig. S15.** mRNA levels of SRM **a** and SMS **b** in fibroblasts with different ages, normalized to GAPDH mRNA. (n=3 per group). Data are means ± SD of biologically independent samples. Statistical significance was calculated using an ordinary one-way ANOVA (*p＜0.05; **p＜0.01; ***p ＜0.001; ****p＜0.0001).


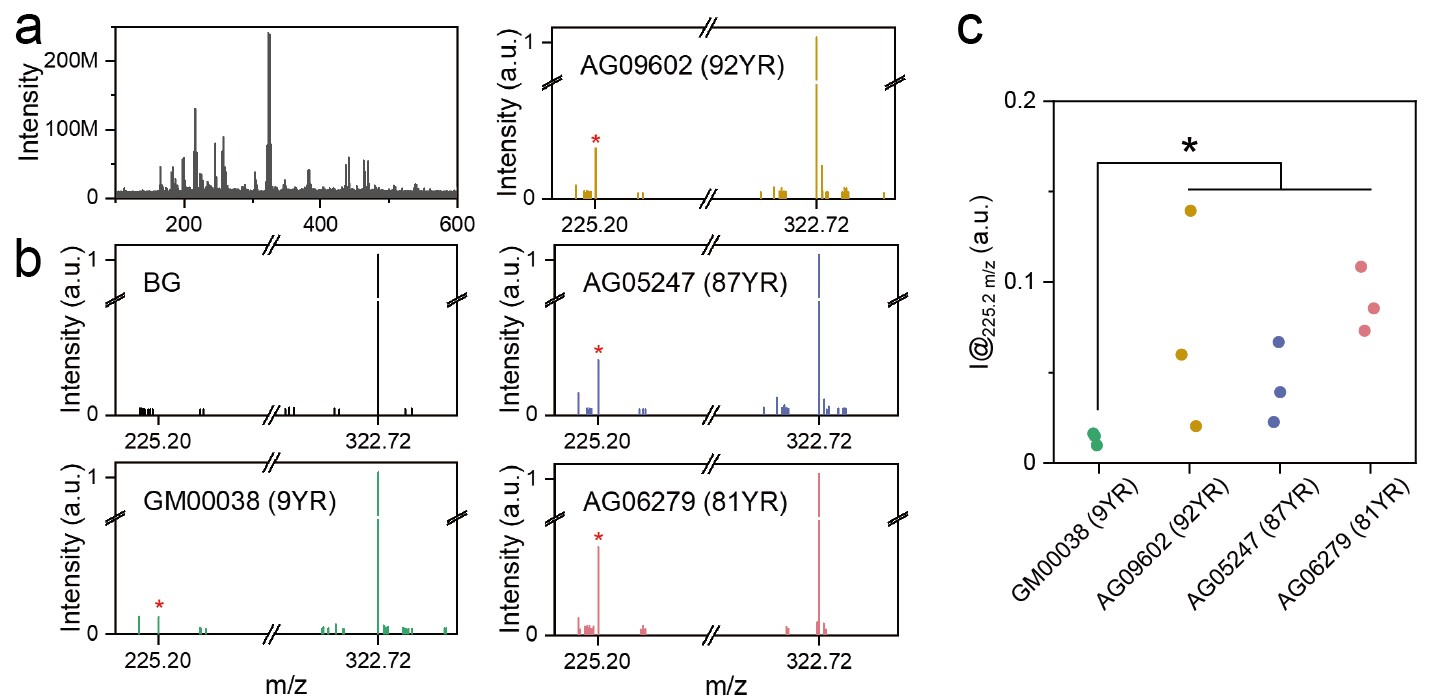


**Fig. S16.** Mass spectrometry validation of the accumulation of spermine in aged cells. **a** A representative mass spectrum of fibroblasts. **b** Comparison of the mass spectra of Ag NPs background, young cells and aged cells, * indicating the spectral peaks corresponding to spermine positive ion patterns. **c** The relative content of spermine in young and aged cells was determined by mass spectrometry. n = 3 per group. Data are means ± SD. Statistical significance was calculated using an ordinary one-way ANOVA (*p＜0.05).


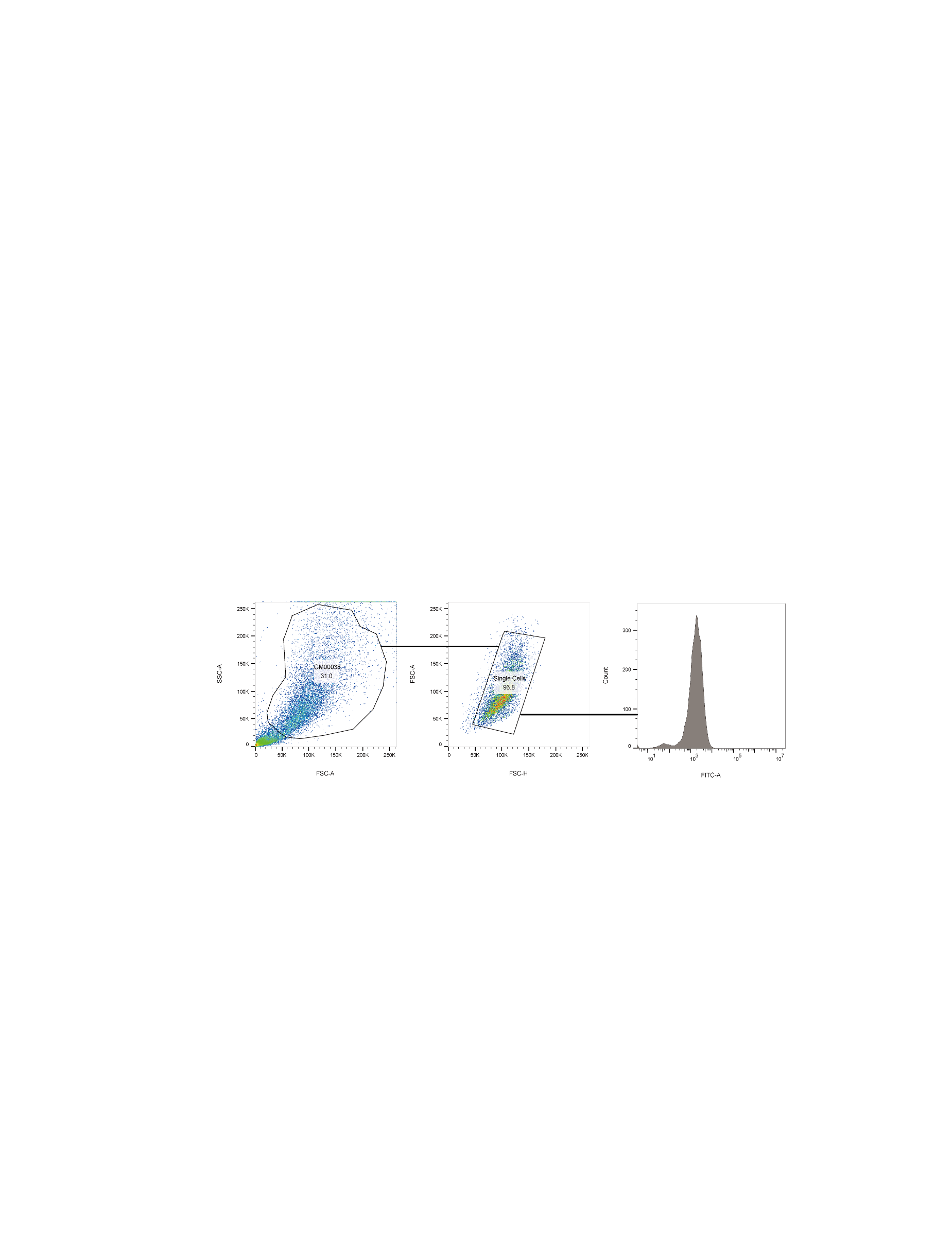


**Fig. S17.** Representative gating strategy used for ΔMFI analysis. A preliminary FSC/SSC gate was implemented to omit cell debris and a secondary FSC-A/FSC-H gate was used to exclude adhesion cells. The single-cell population was used to quantify MFI.

**Table S1.** Information of 20 pure metabolites.

| **Name** | **Abbreviation** | **Source** | **Specifications** |
| --- | --- | --- | --- |
| Spermine | Spm | Sigma-Aldrich | ≥96% |
| N-Acetyl-L-Aspartic Acid | NAA | Bidepharm | 95% |
| Isoleucine | Ile | Aladdin | 99% |
| Taurine | Tau | Yuanye | 99%, BR |
| Xanthine | Xan | Rhawn | 99.50% |
| L-Carnosine | Car | Yuanye | 98%, BR |
| Guanine | Gua | Aladdin | 99% |
| L-Glutamine | Gln | Aladdin | 99% |
| Adenine | Ade | Macklin | ≥99.5% (HPLC) |
| Homocysteine | Hcy | Sigma-Aldrich | >95% |
| Pyridoxine | VB6 | Rhawn | 98% |
| Serotonin | 5-HT | Rhawn | 100%, AR |
| Acetylcholine Chloride | AChCl | Macklin | 99% |
| Choline Chloride | ChCl | Aladdin | 98%, AR |
| Hypoxanthine | Hyp | Aladdin | 99% |
| Cystine | Cys | Sigma-Aldrich | >95% |
| Nicotinic Acid | VB3 | Yuanye | ≥ 99% |
| Sodium Lactate | NaL | Sigma-Aldrich | ~98% |
| Uracil | Ura | Aladdin | 98% |
| Cysteamine | CA | Sigma-Aldrich | >95% |

**Table S2.** Primers for human genes in real-time PCR.

| **Gene** | **Forward (5’→3’)** | **Reverse (5’→3’)** |
| --- | --- | --- |
| **GAPDH** | CCATGGGTGGAATCATATTGGA | TCAACGGATTTGGTCGTATTGG |
| **P16** | GTCGGGTAGAGGAGGTGCG | CATGACCTGGATCGGCCTC |
| **P21** | TCACTGTCTTGTACCCTTGTGC | CCGTTTTCGACCCTGAGAG |
| **LMNB1** | GAAAAAGACAACTCTCGTCGCA | GTAAGCACTGATTTCCATGTCCA |
| **IL-6** | GCCCAGCTATGAACTCCTTCT | GAAGGCAGCAGGCAACAC |
| **IL-8** | AGACAGCAGAGCACACAAGC | ATGGTTCCTTCCGGTGGT |
| **IL-1A** | CATTGGCGTTTGAGTCAGCA | CATGGAGTGGGCCATAGCTT |
| **SRM** | CACCGGGTCCAGTGTGAGATCGACG | AAACCGTCGATCTCACACTGGACCC |
| **SMS** | CACCGACTTACTAACATCCCCACTA | AAACTAGTGGGGATGTTAGTAAGTC |
| **SMOX** | GACTTACTTCCCCGGCTCAG | GTGGCGTGTCCAAGTTTCAC |
| **SSAT** | CTCCGGAAGGACACAGCATT | ACCTCATTGCAACCTGGCTT |
